# Safety and Efficacy of Outpatient Parenteral Antibiotic Therapy (OPAT) in Patients With Infective Endocarditis: A Systematic Review and Meta‐Analysis

**DOI:** 10.1002/clc.70147

**Published:** 2025-05-14

**Authors:** Hamza Ashraf, Zain Ali Nadeem, Khawaja Abdul Rehman, Shanzay Akhtar, Haider Ashfaq, Muhammad Sohaib Khan, Mahad Butt, Ibrahim Nagmeldin, Eeshal Fatima, Muhammad Waqas, Aalaa Saleh, Hritvik Jain, Raheel Ahmed

**Affiliations:** ^1^ Department of Medicine Allama Iqbal Medical College Lahore Pakistan; ^2^ Department of Medicine CMH Lahore Medical College Lahore Pakistan; ^3^ Department of Medicine Karachi Medical and Dental College Karachi Pakistan; ^4^ Department of Medicine University of Khartoum Khartoum Sudan; ^5^ Department of Medicine Services Institute of Medical Sciences Lahore Pakistan; ^6^ Department of Medicine Wah Medical College Wah Pakistan; ^7^ Faculty of Medicine Lebanese University Beirut Lebanon; ^8^ Department of Cardiology All India Institute of Medical Sciences (AIIMS) Jodhpur India; ^9^ National Heart and Lung Institute Imperial College London London UK

**Keywords:** antibiotics, infective endocarditis, OPAT, outpatient, parenteral therapy

## Abstract

**Background:**

Infective endocarditis (IE) is a life‐threatening infection requiring prolonged intravenous antimicrobial therapy. Outpatient parenteral antibiotic therapy (OPAT) has emerged as an alternative to prolonged hospitalization, but its safety and efficacy in IE remain debated.

**Hypothesis:**

This systematic review and meta‐analysis aimed to evaluate the outcomes of OPAT in IE patients.

**Methods:**

We systematically searched MEDLINE, Cochrane CENTRAL, Google Scholar, and Scopus for studies assessing OPAT in IE. Eligible studies included randomized controlled trials and observational studies reporting at least one relevant outcome (mortality, relapse, readmission, valve surgery, and adverse events). Pooled estimates were calculated using a random‐effects model, and heterogeneity was assessed using the I² statistic. Risk of bias was evaluated using the ROBINS‐I tool.

**Results:**

A total of 25 studies involving 2654 patients were included in the analysis. Patients treated with OPAT had a mortality rate of 0% during the treatment period and 5% during follow‐up. The readmission rate was 16% during the treatment period, 4% of the patients had relapse, while 16% of patients underwent cardiac surgery. During follow‐up, the readmission rate was 19%, with a relapse rate of 2%, and 14% of patients underwent cardiac surgery. Sensitivity analyses did not significantly affect the results, highlighting the robustness of the findings.

**Conclusion:**

OPAT appears to be safe and effective for IE patients, with low mortality and relapse rates. However, increased readmission rates and IV‐line complications warrant careful patient selection and monitoring. Further prospective trials are needed to refine OPAT protocols.

## Summary

- Outpatient parenteral antibiotic therapy (OPAT) is an effective and safe treatment option for infective endocarditis, with favorable mortality, relapse, and readmission outcomes during both treatment and follow‐up periods.
- Future research should focus on randomized controlled trials to directly compare OPAT with inpatient care, refine patient selection criteria, and enhance monitoring strategies to optimize outcomes.

## Graphical abstract

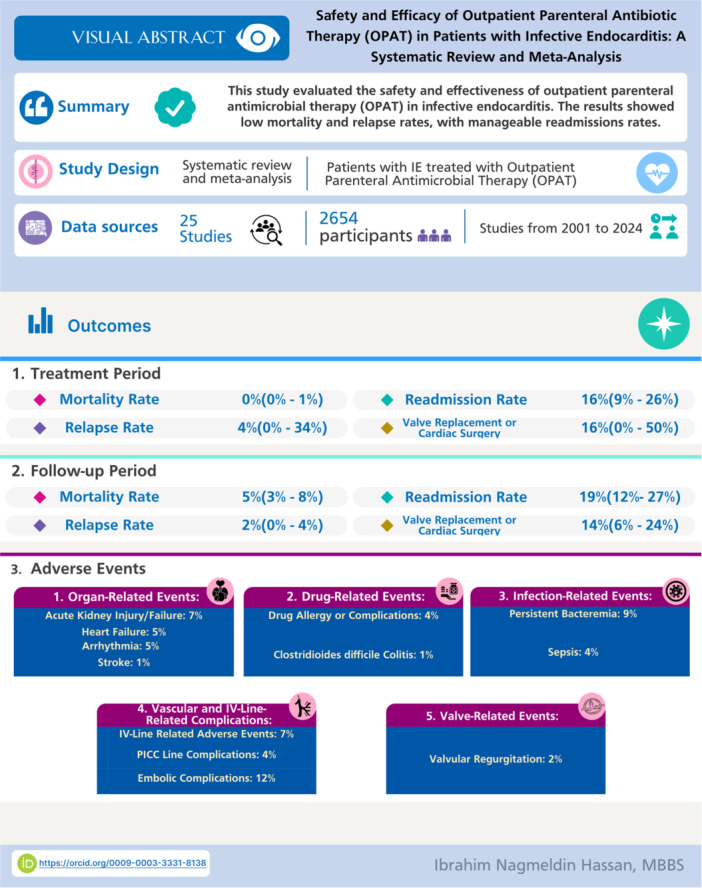

**Abbreviations**
AKI: acute kidney injury
IE: infective endocarditis
OPAT: outpatient parenteral antibiotic therapy
RCT: randomized controlled trial

## 1 Introduction

Infective endocarditis (IE) is a rare but severe infection affecting the heart's inner lining and intracardiac devices. Globally, its incidence ranges from 3 to 10 cases per 100,000 people annually, with an increasing trend due to an aging population and the rise in invasive medical procedures [1]. Despite its infrequency, IE is associated with high morbidity and mortality, with hospital mortality rates between 15% and 30% [1, 2]. Management is challenging, requiring prolonged intravenous antimicrobial therapy—typically 4–6 weeks—to ensure effective treatment and prevent antimicrobial resistance [3]. However, extended hospitalization increases the risk of hospital‐acquired infections, particularly in elderly and frail patients who constitute a significant proportion of IE cases [4, 5]. Additionally, prolonged hospital stays negatively impact quality of life and contribute to substantial healthcare costs [6].

Outpatient parenteral antimicrobial therapy (OPAT) offers a promising alternative to traditional hospital‐based treatment, allowing intravenous antibiotics to be administered at home or in outpatient settings. This approach reduces hospitalization time, frees up hospital beds, lowers costs, and enhances patient satisfaction [7, 8]. However, OPAT is not without risks, including adverse drug reactions, complications related to intravenous access, and unexpected clinical deterioration [9]. Bloodstream infections associated with intravenous catheters are of particular concern in IE patients, as they can increase mortality risk [10].

Previous meta‐analyses on OPAT for IE have been limited, and several new studies have emerged since their publication [11]. Moreover, prior analyses, though comprehensive, did not include patients who underwent cardiac surgery—a significant subset of IE cases. To address these gaps and prevailing uncertainties, we conducted an updated meta‐analysis to evaluate the efficacy and safety of OPAT in patients with IE.

## 2 Methods

This systematic review and meta‐analysis conform to the standards established by Cochrane [12] and the Preferred Reporting Items for Systematic Reviews and Meta‐Analyses (PRISMA) guidelines [13]. The PRISMA checklist is provided in Supporting Information S1: Table 1. This review is registered with the International Prospective Register of Systematic Reviews (PROSPERO: CRD42024557360). Ethical approval was not required, as the analysis was conducted using pre‐existing published data.

### 2.1 Data Sources and Search Strategy

Two reviewers conducted a comprehensive search for eligible studies from inception until June 2024 using the following electronic databases: MEDLINE (via PubMed), Cochrane CENTRAL Library, Google Scholar, and Scopus. The detailed search strings for each database are outlined in Supporting Information S1: Table 2. No search filters were applied for study type. There were no limitations based on country or race. The reference lists of the included studies were manually reviewed to identify any studies that might have been missed during the search.

### 2.2 Study Selection and Eligibility

Studies were included if they met the following eligibility criteria: (a) patients diagnosed with IE, (b) use of OPAT as the intervention, and (c) reporting at least one of the following outcomes during treatment or follow‐up—readmission, all‐cause mortality, IE relapse, valve replacement/cardiac surgery, and complications such as drug allergy, acute kidney injury, and sepsis. (d) Observational studies, including retrospective and prospective cohort studies, as well as randomized controlled trials (RCTs), were considered.

Conversely, studies were excluded if they did not focus on OPAT in infective endocarditis, lacked accessible outcome data, presented duplicate information or overlapping participants, or were reviews, editorials, book chapters, letters, expert opinions, conference papers, case reports, or animal studies.

### 2.3 Data Extraction

Articles obtained from the systematic search were transferred to EndNote Reference Library software to identify and remove duplicates. The remaining articles were then reviewed by two independent reviewers, and only those meeting the predetermined criteria were included. The initial screening involved selecting studies based on their titles and abstracts, followed by a thorough review of the full‐text articles to ensure their relevance. Any conflicts were resolved through consensus or by consulting a third reviewer.

Data extracted from the studies included: (1) study characteristics, such as the first author, publication year, country, and study design; (2) patient demographics, including the number of patients, mean age, and sex ratio; (3) disease characteristics, such as type of endocarditis and diagnosis according to Duke's criteria; (4) treatment details, including OPAT duration, mean inpatient length of stay, and follow‐up time; and (5) outcomes and complications. If data for multiple outcomes were reported collectively, the corresponding authors were contacted via email to request separate results.

### 2.4 Quality Assessment

The risk of bias was assessed using the Risk of Bias in Non‐randomized Studies of Interventions (ROBINS‐I) tool [14]. Two authors independently assessed the studies based on the tool's criteria. Any discrepancies were resolved by discussion.

In each study, bias was assessed in seven domains: confounding, selection of participants, classification of interventions, deviations from intended interventions, missing outcome data, outcome measurement, and selection of reported results. Bias in each domain was categorized as low, moderate, serious, or critical. Finally, the prior assessment results were used to determine the overall risk of bias in each study.

### 2.5 Statistical Analysis

The statistical analysis was conducted in R version 4.4.1 using the “meta” and “metasens” packages. A random‐effects model was used to compute pooled proportions for dichotomous outcomes. To stabilize variances, the Freeman‐Tukey double arcsine transformation was applied before pooling using the inverse variance method [15]. The Hartung‐Knapp adjustment was incorporated to improve the accuracy of confidence intervals by accounting for between‐study variability [16]. Forest plots were created to visually represent the results.

Heterogeneity was evaluated using the Higgins I² statistic, following predefined thresholds from the *Cochrane Handbook of Systematic Reviews of Interventions*: 0%–40%: low heterogeneity; 30%–60%: moderate heterogeneity; 50%–90%: substantial heterogeneity; and 75%–100%: considerable heterogeneity [17].

Publication bias was assessed using funnel plots and Egger's test [18] when an outcome was reported in at least 10 studies. To ensure robustness, sensitivity analysis was performed using the leave‐one‐out approach. A two‐tailed *p*‐value < 0.05 was considered statistically significant in all instances.

## 3 Results

**Figure 1:**
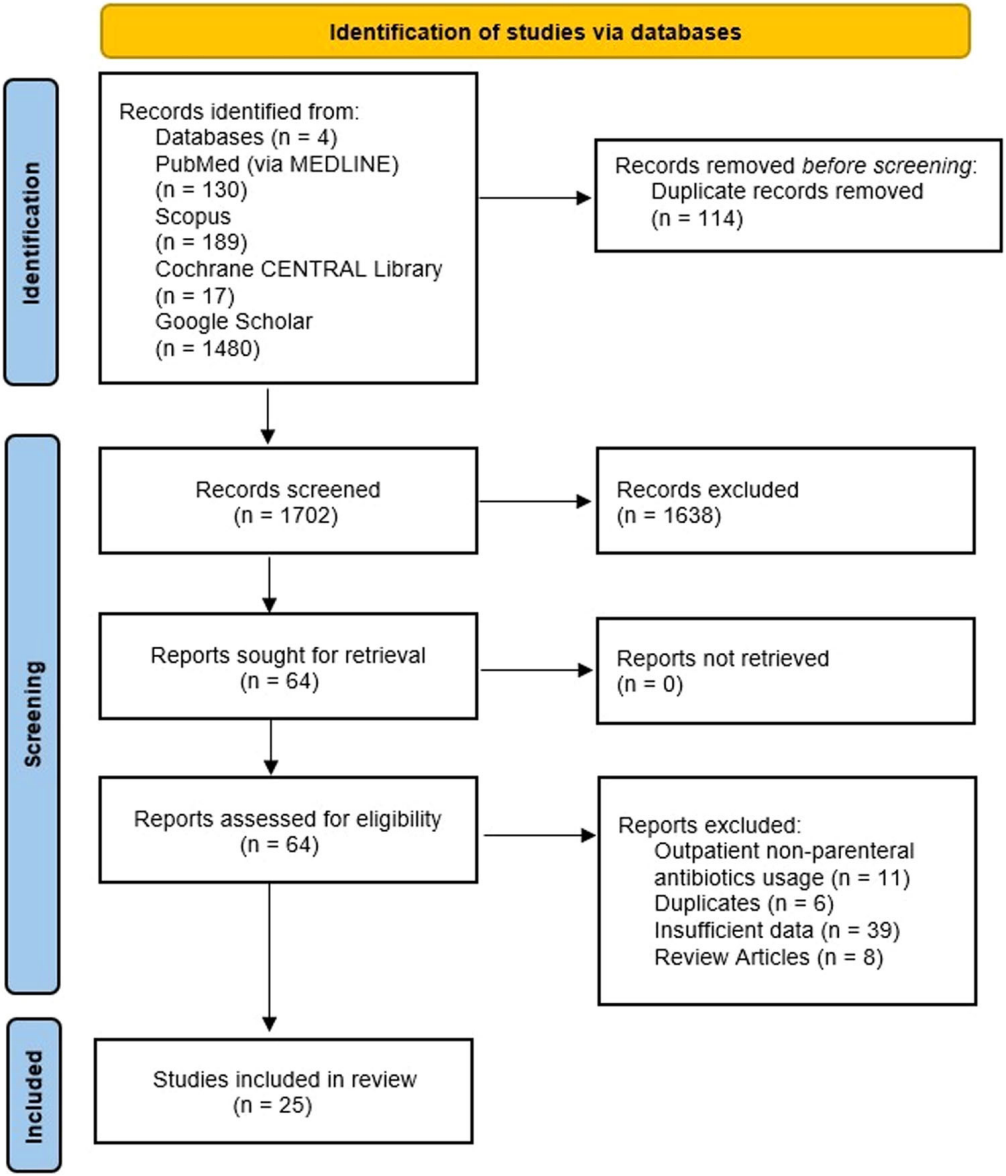
PRISMA flow chart of included literature.

We identified 1816 articles from our electronic search, 114 of which were duplicates. After screening based on titles, abstracts, and then full texts, 25 studies [19, 20, 21, 22, 23, 24, 25, 26, 27, 28, 29, 30, 31, 32, 33, 34, 35, 36, 37, 38, 39, 40, 41, 42]—19 retrospective, five prospective, and one ambispective—involving 2654 patients were included in this systematic review and meta‐analysis. Figure 1 shows the PRISMA flow diagram for the study selection process. The included studies were published from 2001 to 2024. Most of the patients were male and had native valve endocarditis. The OPAT duration and mean follow‐up period varied across the studies. Table 1 shows the baseline characteristics of the included studies.

**Table 1:** Baseline characteristics of included studies.

Study	Country	Study Design	Total Patients (n)	Age in Years (mean ± SD)	Sex (M:F)	Type of endocarditis (%)	OPAT duration in days (mean ± SD)	Diagnosis according to Duke's criteria	Most common affected valve (%)	In‐patient length of stay in days (mean ± SD)	Follow‐up time
Kortajarena et al. [19]	Spain	Prospective	194	65 (25–92)^c^	140:54	Native valve (49.5%), Prosthetic valve (31.4%), Electrocatheter (10.3%), Unknown (8.8%)	22.3 ± 14.4	Definite or Possible IE	Aortic Valve (42.3%)	16 ± 10.2	NR
Pericàs et al. [20]	Spain	Prospective	429	67.8 (55.9–76.4)^a^	303:126	Native valve (57.1%), Prosthetic valve (27.3%), Pacemaker/defibrillator (18.6%)	42.0 (32–54)^a^	Definite or Possible IE	Aortic Valve (43.8%)	NR	1 year
Durojaiye et al. [21]	UK	Retrospective	146	60 ± 17.6	131:15	Native valve (68.5%), Prosthetic valve (31.5%)	23 (17–31)^a^	Definite or Possible IE	Aortic valve (42.3%)	21 (16–30)^a^	1 year
Htin et al. [22]	Australia	Retrospective	68	68 (21–93)^b^	59:9	Native valve (43%), Prosthetic valve (35%), Pacemaker/defibrillator (19%), Others (3%)	24 (4–42)^b^	Definite or Possible IE	Aortic valve (50%)	NR	1 year
Pajarón et al. [23]	Spain	Ambispective	54	61 ± 16.5	43:11	Native valve (64.9%), Prosthetic valve (35.1%)	21.7 ± 6.3	Definite or Possible IE	Aortic valve (42.1%) Mitral valve (42.1%)	49	1 year
Partridge et al. [24]	UK	Retrospective	34	54.7 (16–82)^c^	27:7	Native valve (61.1%), Prosthetic valve (30.6%), Other (8.3%)	27 (7–65)^b^	Definite or Possible IE	Mitral valve (44.4%)	23^d^	30 (6–57)^b^ months
Cervera et al. [25]	Spain	Prospective	73	59.5 ± 18.7	55:18	Native valve (58%), Prosthetic valve (32%), Pacemaker‐lead (10%)	17 (11–26.5)^a^	Definite, Possible, or Probable IE	Mitral valve (47%)	21 (13–29)^a^	1 year
Lacroix et al. [26]	France	Retrospective	18	59.5 (17–86)^c^	11:7	Prosthetic valve (50.0%), Others (50.0%)	NR	All Definite IE	Aortic valve (44.4%) Mitral valve (44.4%)	23.5 (8–55)^c^	3 months
Amodeo et al. [27]	New Zealand	Prospective	94	64.5 (24–94)^c^	75:25	NR	20.5 (2‐49)^b^	Definite or Possible IE	Aortic valve (28%) Mitral valve (28%)	14 (2–42)^b^	1 year
Aparicio‐Minguijón et al. 2024	Spain	Retrospective	61	78.5 (63.2–85.2)^a^	48:13	Native valve (49.2%), Prosthetic valve (41%), Pacemaker/ICD (9.8%)	47 (42–57.5)^a^	All Definite IE	Mitral valve (32.8%)	27 (20–34)^a^	6 months
Camazon et al. [28]	Spain	Retrospective	8	57.1 ± 23.6	7:1	Prosthetic valve (62.5%), Others (37.5%)	56 (42 − 84)^a^	All Definite IE	Aortic valve (50%)	NR	3 years
Campbell et al. [29]	New Zealand	Retrospective	115	66 (19–85)^b^	89:26	Native valve (72%), Prosthetic valve (28%)	27 (19–35)^a^	Definite or Possible IE	Mitral valve (33%)	12 (8–16)^a^	1 year
Douiyeb et al. [30]	Netherlands	Retrospective	11	NR	NR	NR	14 (8–32)^a^	NR	NR	NR	NR
Freling et al. [31]	United States	Retrospective	211	55 (42–65)^a^	152:59	NR	23 (5–33)^a^	Definite or Possible IE	Aortic Valve (22.3%)	16 (10–31)^a^	204 (51–495)^a^ days
Garcia‐Carretero et al. [32]	Spain	Retrospective	26	66.5 ± 14.1	23:3	Native valve (57.7%), Prosthetic valve (42.3%)	31.0 ± 12.4	Definite or Possible IE	Mitral valve (50%)	10.5 ± 6.3	1 year
Hamad et al. [33]	United States	Retrospective	276	NR	NR	NR	15 (7–28)^a^	NR	NR	7 (5–12)^a^	90 days
Herrera‐Hidalgo et al. [34]	Spain	Retrospective	27	70.2 ± 15.7	19:8	Native valve (52%), Prosthetic valve (37%), Other (11%)	NR	Definite or Possible IE	Aortic valve (44.4%)	22.8 ± 10.2	6–12 months
Ingram et al [35]	Australia	Retrospective	20	69 (60–79)^a^	14:6	Native valve (55%), Prosthetic valve (35%), Cardiac‐related devices (10%)	22 (8–34)^a^	All Definite IE	Aortic valve (65%)	17 (12–22)^a^	1 year
Kwok et al. [36]	England	Retrospective	13	72.5 ± 14.1	8:5	Native valve (54%), Prosthetic valve (46%)	21 (6–26)^c^	NR	Aortic valve (54%)	22	NR
Pericàs et al. 2022	Spain	Prospective	558	69 (57–77)^a^	376:182	Native valve (56.5%), Prosthetic valve (25.9%), Cardiovascular implantable electronic devices (17.6%)	49 (3.9)^a^	Definite or Possible IE	Aortic valve (45.5%)	18 (13–29)^a^	1 year
Schweibert et al. [37]	UK	Retrospective	101	68 (18–92)^b^	70:31	Native valve (62%), Prosthetic valve (28%), Intracardiac device‐related (8%), Other (2%)	12 (1–59)^b^	Definite or Possible IE	Aortic valve (41%)	27 (6–94)^b^	1 year
Suárez et al. 2023	Spain	Retrospective	22	74 (59–82)^a^	15:7	Prosthetic valve (64%), Native valve (36%)	42 (42–49)^a^	All Definite IE	Aortic valve (68%)	22 (16–34)^a^	1 year
Gil‐Navarro et al. 2017	Spain	Retrospective	4	72 ± 12.78	4:0	Native valve = 4 (100%)	22.5 (13–32)^a^	NR	Aortic valve (50%)	25 (15–32)^a^	365 (221–406)^a^ days
Larioza et al. [38]	United States	Retrospective	43	Age < 50: *n* = 19; Age > 50: *n* = 24	29:14	Native valve (74%), Prosthetic valve (19%), Other (7%)	NR	Definite, Possible, or Probable IE	Mitral valve (42%)	NR	1 year
Lopardo et al. [39]	Argentina	Retrospective	48	55 (17–76)^b^	30:18	Native valve (87.5%), Prosthetic valve (12.5%)	NR	Definite or Possible IE	NR	NR	NR

Abbreviations: F, female; IE, infective endocarditis; M, male; n, number of participants; NR, not reported; OPAT, outpatient parenteral antibiotic therapy; SD, standard deviation.

^a^ = Median (Interquartile range).

^b^ = Median (range).

^c^ = Mean (range).

^d^ = Median.

### 3.1 Risk of Bias

Twenty‐five studies were assessed for risk of bias [19, 20, 21, 22, 23, 24, 25, 26, 27, 28, 29, 30, 31, 32, 33, 34, 35, 36, 37, 38, 39, 40, 41, 42]. Eight studies (32%) (Cervera 2011 [25], Durojaiye 2021 [21], Kortajarena 2017 [19], Lacroix 2017 [26], Herrera‐Hidalgo L 2021 [34], Pericás JM 2022 [20], Schwiebert R 2023 [37], and Suárez M 2023 [42]) had a serious risk of bias, while the other 17 studies (68%) were rated with a moderate risk (Supporting Information S1: Table 3). The results of the quality assessment were presented visually in the form of a traffic light plot and summary plot using the risk of bias visualization (Robvis) tool (Supporting Information S1: Figure 1). The ROBINS‐I domains that most contributed to moderate or serious risk of bias were confounding bias (D1), participant selection bias (D2), bias due to intervention protocol deviations (D4), and bias in the measurement of outcomes (D6).

### 3.2 Results of the Meta‐Analysis

Table 2 depicts the results of the meta‐analysis in detail.

**Table 2:** Summary of meta‐analysis results for OPAT outcomes and adverse events.

Treatment Period					
Outcome	No. of Studies	Events/Total	Pooled Proportion	95% CI	I^2^
**Mortality**	10	8/563	0	0.00–0.01	0%
**Relapse**	2	5/122	0.04	0.00–0.34	0%
**Readmission**	11	118/696	0.16	0.09–0.26	86%
**Cardiac Surgery/Valve Replacement**	6	66/613	0.16	0.00–0.50	95%
**Follow‐up Period**					
**Mortality**	20	159/2238	0.05	0.03–0.08	75%
**Relapse**	14	46/1786	0.02	0.00–0.04	56%
**Readmission**	10	372/1907	0.19	0.12–0.27	92%
**Cardiac Surgery/Valve Replacement**	10	195/1574	0.14	0.06–0.24	92%
**Adverse Events**					
**Drug Allergy/Drug complication**	13	40/895	0.04	0.02–0.06	43%
**Acute Kidney Injury/Failure**	6	135/969	0.07	0.01–0.17	95%
**IV‐line Related Adverse Events**	5	55/653	0.07	0.02–0.16	80%
**Clostridioides Difficile Colitis**	4	6/411	0.01	0.00–0.03	0%
**PICC Line Related Complications**	7	26/557	0.04	0.00–0.10	78%
**Valvular Regurgitation**	3	6/287	0.02	0.00–0.23	69%
**Heart Failure**	10	34/767	0.05	0.02–0.10	65%
**Arrythmia**	3	32/503	0.05	0.00–0.13	8%
**Stroke**	3	3/232	0.01	0.00–0.06	0%
**Persistent Bacteremia**	2	91/987	0.09	0.01–0.24	0%
**Sepsis**	4	43/1114	0.04	0.02–0.05	0%
**Embolic Complications**	6	378/1455	0.12	0.00–0.33	98%

#### 3.2.1 Treatment Period

**Figure 2:**
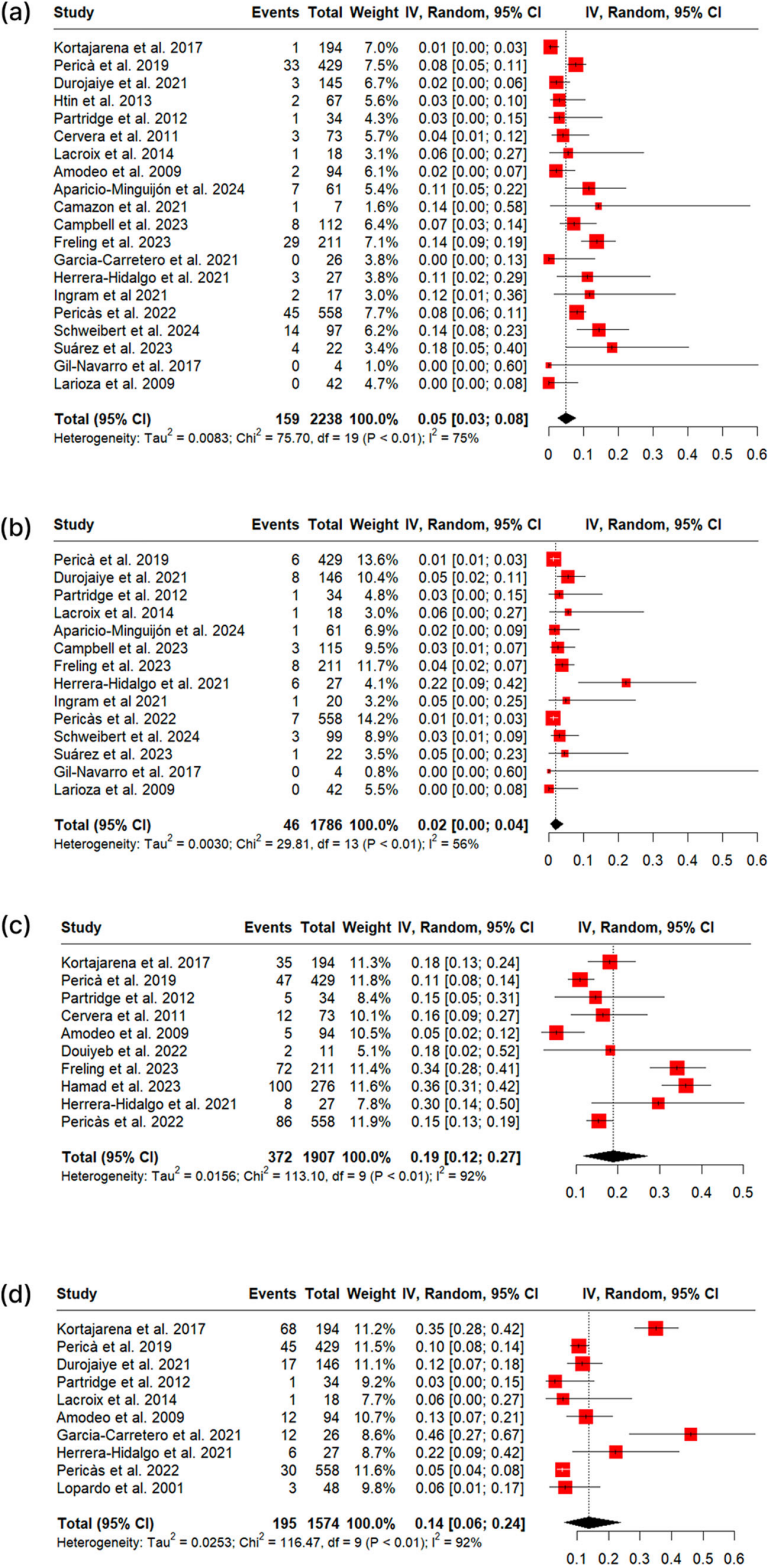
OPAT outcomes during the treatment period; Figure (2a) shows the mortality rate; Figure (2b) shows the relapse rate; Figure (2c) shows the readmission rate; Figure (2d) shows the percentage of patients requiring valve replacement or cardiac surgery.

**Figure 3:**
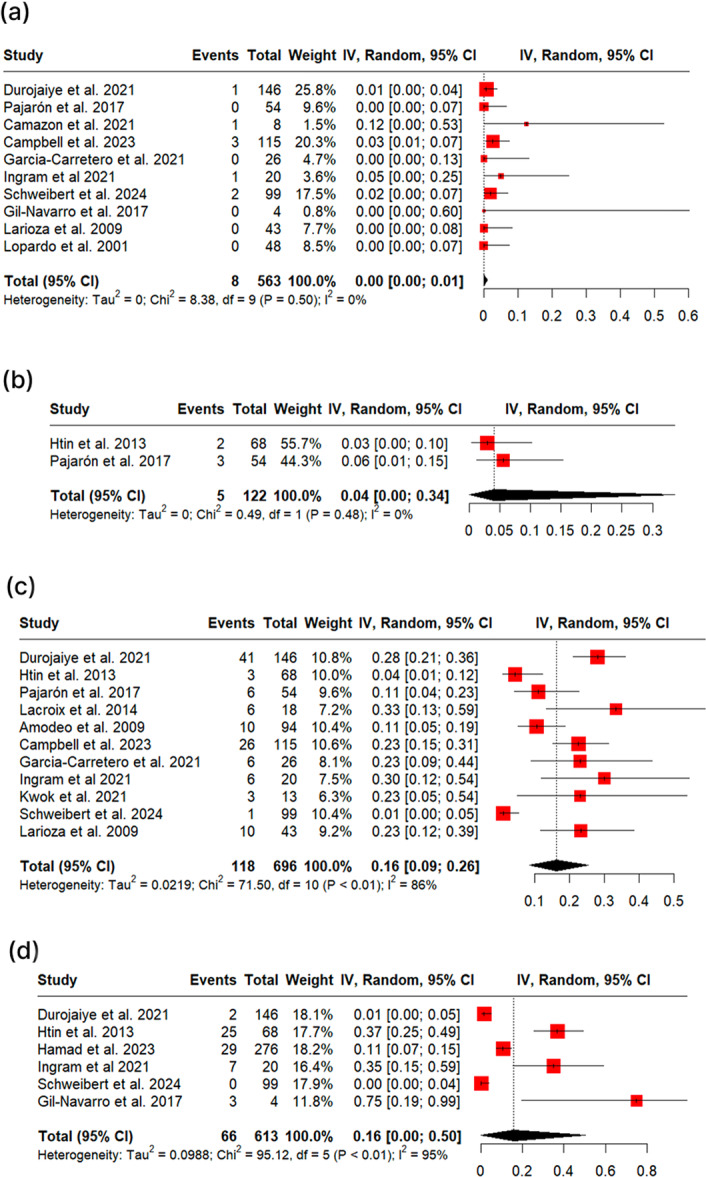
Clinical outcomes during the follow‐up period for patients treated with OPAT; Figure (3a) shows the mortality rate; Figure (3b) shows the relapse rate; Figure (3c) shows the readmission rate; Figure (3d) shows the percentage of patients requiring valve replacement or cardiac surgery.

During the treatment period, patients treated with OPAT showed a mortality rate of 0% (95% CI: 0%–1%, I² = 0%, Figure 2a), relapse rate of 4% (95% CI: 0%–34%, I² = 0%, Figure 2b), readmission rate of 16% (95% CI: 9%–26%, I² = 86%, Figure 2c), and 16% (95% CI: 0%–50%, I² = 95%, Figure 2d) of the patients required valve replacement or cardiac surgery. Sensitivity analysis, omitting one study at a time, did not impact the results much (Supporting Information S1: Figures 2–5).

#### 3.2.2 Follow‐Up Period

During the follow‐up period, patients treated with OPAT showed a mortality rate of 5% (95% CI: 3%–8%, I² = 75%, Figure 3a), relapse rate of 2% (95% CI: 0%–4%, I² = 56%, Figure 3b), readmission rate of 19% (95% CI: 12%–27%, I² = 92%, Figure 3c), and 14% (95% CI: 6%–24%, I² = 92%, Figure 3d) of the patients required valve replacement or cardiac surgery. Sensitivity analysis, omitting one study at a time, did not impact the results much (Supporting Information S1: Figures 6–9), but omitting the study by Herrera‐Hidalgo et al. (2021) [34] reduced the heterogeneity for the relapse rate (1%, 95% CI: 0%–2%, I² = 28%).

#### 3.2.3 Adverse Events

Of the patients treated with OPAT, 4% (95% CI: 2%–6%, I² = 43%, Supporting Information S1: Figure 10) had a drug allergy or complication, 7% (95% CI: 1%–17%, I² = 95%, Supporting Information S1: Figure 11) showed acute kidney injury or failure, 7% (95% CI: 2%–16%, I² = 80%, Supporting Information S1: Figure 12) showed IV‐line related adverse events, 1% (95% CI: 0%–3%, I² = 0%, Supporting Information S1: Figure 13) showed Clostridioides difficile colitis, 4% (95% CI: 0%–10%, I² = 78%, Supporting Information S1: Figure 14) had PICC line complications, 2% (95% CI: 0%–23%, I² = 69%, Supporting Information S1: Figure 15) had valvular regurgitation, 5% (95% CI: 2%–10%, I² = 65%, Supporting Information S1: Figure 16) showed heart failure, 5% (95% CI: 0%–13%, I² = 8%, Supporting Information S1: Figure 17) had an arrhythmia, 1% (95% CI: 0%–6%, I² = 0%, Supporting Information S1: Figure 18) had a stroke, 9% (95% CI: 1%–24%, I² = 0%, Supporting Information S1: Figure 19) had persistent bacteremia, 4% (95% CI: 2%–5%, I² = 0%, Supporting Information S1: Figure 20) had sepsis, and 12% (95% CI: 0%–33%, I² = 98%, Supporting Information S1: Figure 21) had embolic complications. Sensitivity analysis, omitting one study at a time, had minimal impact on the results (Supporting Information S1: Figures 22–33).

#### 3.2.4 Publication Bias

Funnel plot inspection and Egger's test revealed some evidence of publication bias in patients with heart failure (*p* = 0.0038, Supporting Information S1: Figure 34). No publication bias was observed for mortality rate (*p* = 0.2986, Supporting Information S1: Figure 35) and readmission rate (*p* = 0.5806, Supporting Information S1: Figure 36) during the treatment period, for mortality rate (*p* = 0.8285, Supporting Information S1: Figure 37), relapse rate (*p* = 0.0205, Supporting Information S1: Figure 38), readmission rate (*p* = 0.7901, Supporting Information S1: Figure 39), and patients requiring valve replacement or cardiac surgery (*p* = 0.3503, Supporting Information S1: Figure 40) during the follow‐up period, and for patients having a drug allergy or complication (*p* = 0.0834, Supporting Information S1: Figure 41). Remaining outcomes could not be assessed as they were reported in fewer than 10 studies.

## 4 Discussion

This systematic review and meta‐analysis of 25 studies, involving 2654 patients, highlights the viability of outpatient parenteral antimicrobial therapy (OPAT) as a treatment for infective endocarditis (IE), demonstrating low rates of mortality and relapse, consistent with previous research [11]. OPAT allows selected patients to complete intravenous antibiotic therapy outside the hospital, potentially reducing hospital stays and associated costs [6, 43]. However, a significant proportion of patients required readmission or surgical intervention, and complications such as IV‐line issues, renal injury, and embolic events were observed, emphasizing the need for careful patient selection and close monitoring [9, 10].

This updated meta‐analysis expands on the findings of Wen et al. (2022) by incorporating a larger data set (25 vs. 9 studies; 2654 vs. 1116 patients) [11]. Wen et al. [11] reported mortality rates of 4% during treatment and 3% during follow‐up, whereas our study found 0% and 5%, respectively. Relapse rates of 4% and 2%, along with readmission rates of < 20%, were consistent between the two studies. However, the previous meta‐analysis [11] did not assess additional efficacy outcomes, such as the proportion of patients requiring cardiac surgery or valve replacement. Moreover, we performed a leave‐one‐out sensitivity analysis, which was not included in Wen et al.'s [11] study. Additionally, unlike Wen et al. [11], who combined treatment and follow‐up outcomes—potentially leading to statistical overestimation—our study analyzed these outcomes separately. We also found evidence of publication bias for heart failure outcomes, which Wen et al. [11] did not report. Furthermore, unlike the previous meta‐analysis which broadly reported an adverse event rate of 26%, our analysis provides a more detailed breakdown of adverse events, including acute kidney injury, IV‐line complications, persistent bacteremia, sepsis, valvular regurgitation, arrhythmia, and embolic complications, which were not explicitly examined in prior studies. Another meta‐analysis by Mohammed et al. (2024) compared the efficacy and safety profile of OPAT with inpatient treatment in a different clinical population. Their findings showed no significant differences in mortality, treatment failure, or adverse events, concluding that OPAT is a safe and effective alternative to inpatient intravenous antimicrobial therapy [44].

Our analysis revealed that 16% of patients receiving OPAT required readmission during treatment, aligning with previous studies [30], while 19% required readmission during follow‐up. The primary reasons for OPAT‐related readmissions included infection recurrence, adverse drug reactions, and IV‐line complications. Risk factors such as vancomycin use and prolonged OPAT duration further contributed to readmission [45, 46]. Administrative factors, including loss of insurance, also played a role, with patients having multiple comorbidities facing higher readmission risk. Additionally, the COVID‐19 pandemic influenced outcomes by increasing telehealth utilization, which, while reducing missed appointments, may have limited in‐person evaluations necessary for early complication detection [46]. Durojaiye et al. identified an elevated Charlson Comorbidity Index as a predictor of OPAT failure due to worsening clinical conditions necessitating inpatient care [21]. Readmission places a burden on both patients and healthcare systems, reducing resources available for critically ill individuals [6, 47, 48].

Our findings also indicate that 16% of OPAT patients required valve replacement or cardiac surgery during treatment, and 14% required surgery during follow‐up. This underscores the limitations of antibiotic therapy alone in managing certain cases of IE. Surgery was required for persistent infection, valve damage, heart failure, or complications such as abscess formation [4]. Patients with prosthetic valves or highly virulent pathogens were at higher risk for surgical intervention [49]. In some cases, advanced valve dysfunction or recurrent infection necessitated surgery even after completing OPAT [11, 23]. The 2023 ESC guidelines recommend surgery for IE in cases of heart failure due to severe valve dysfunction, uncontrolled infection (persistent sepsis, abscess, or fungal/multidrug‐resistant infection), and prevention of embolization in patients with large vegetations (≥ 10 mm) after embolic events. With surgical urgency categorized into emergency (within 24 h), urgent (within 3–5 days), and elective procedures, all managed by a multidisciplinary endocarditis team for optimal outcomes [50].

Despite its advantages, OPAT is associated with adverse events. The most frequently reported complications in our study were embolic events (12%), persistent bacteremia (9%), acute kidney injury (7%), and IV‐line complications (7%). Patients receiving prolonged antibiotic therapy, particularly those with intravenous drug use (IVDU), were at increased risk for IV‐line complications, including catheter blockage, dislodgement, and catheter‐related bloodstream infections (CRBSI) [51, 52, 53, 54, 55, 56, 57]. Strengthening infection control protocols and improving care coordination can mitigate these risks [58]. Nephrotoxicity, particularly from aminoglycosides and vancomycin, is a well‐documented complication of IE treatment [59]. The observed rates of acute kidney injury in our study align with prior research, which has linked AKI to prosthetic valve infections, acute heart failure, and vancomycin exposure [60]. The combination of vancomycin with piperacillin‐tazobactam significantly increases nephrotoxicity risk due to cumulative renal stress and tubular injury. While AKI is commonly identified in hospitalized patients, its occurrence in OPAT settings remains a concern due to less frequent monitoring. Given its impact on hospital stay length, improved prevention and monitoring strategies are crucial [61].

Persistent bacteremia in OPAT patients suggests bacterial survival mechanisms, such as biofilm formation, particularly with *Staphylococcus aureus* and Enterococcus spp., which complicate eradication, especially in prosthetic valve infections [62]. Embolic events (EEs), including stroke, splenic infarction, and pulmonary embolism, result from vegetation dislodgement. Risk factors for EE include vegetation size, mobility, and valve location, with larger and more mobile mitral valve vegetations posing the highest risk [63]. Inflammatory and clotting indicators such as CRP, D‐dimer, and platelet functionality also play a role in embolization. Recurrent EE may occur as a result of ongoing infection, insufficient treatment, or underlyinpatient characteristics such as past embolism, particular pathogens (e.g., *S. aureus*, Streptococcus bovis), and the host's immune response [63].

Enhanced monitoring and follow‐up for OPAT patients with IE is essential. Regular assessments of vital signs, laboratory parameters, and potential complications can help reduce adverse outcomes. For individuals undergoing OPAT at home or in community facilities, it is essential to create a strong monitoring system [6]. Additionally, the establishment of specialized OPAT teams can further improve patient safety and treatment efficacy. Overall, the benefits of OPAT for IE treatment outweigh its risks when appropriate patient selection and monitoring strategies are implemented.

### 4.1 Study Limitations

This meta‐analysis presents various limitations that need to be taken into account. First, significant heterogeneity was observed in key outcomes, including mortality, relapse, and readmission rates. Variability in study design, patient comorbidities, follow‐up durations, and treatment approaches likely contributed to this heterogeneity. However, we performed a leave‐one‐out sensitivity analysis to address this issue. Second, inconsistencies in the data across included studies limited our ability to perform subgroup analyses based on factors such as sex, age, and comorbidities, which may influence outcomes. Third, the absence of a direct control group (i.e., standard inpatient care) limits our ability to draw definitive comparisons between OPAT and hospitalization. While our results support OPAT's safety and efficacy, conclusions regarding its superiority or non‐inferiority to inpatient care should be made cautiously. Finally, the use of real‐world data introduces potential biases, including confounding factors, missing data, and treatment adherence bias. Since no direct randomized controlled trials (RCTs) compare OPAT with inpatient treatment for IE, our findings rely exclusively on observational data, limiting causal inferences.

## 5 Conclusion

This systematic review and meta‐analysis demonstrate that OPAT is an effective and safe treatment option for IE, with favorable mortality, relapse, and readmission outcomes during both treatment and follow‐up periods. However, future research should focus on randomized controlled trials to directly compare OPAT with inpatient care, refine patient selection criteria, and enhance monitoring strategies to optimize outcomes.

## Author Contributions

Conceptualization: Hamza Ashraf. Data curation: Hamza Ashraf. Muhammad Sohaib Khan, Khawaja Abdul Rehman, and Mahad Butt. Formal Analysis: Zain Ali Nadeem. Methodology: Hamza Ashraf. Project administration: Hamza Ashraf, Haider Ashfaq, and Raheel Ahmed. Validation: Hamza Ashraf. Visualization: Ibrahim Nagmeldin. Writing – original draft: Hamza Ashraf, Zain Ali Nadeem, Khawaja Abdul Rehman, Shanzay Akhtar, Haider Ashfaq, Eeshal Fatima, Muhammad Sohaib Khan, Mahad Butt, Ibrahim Nagmeldin, and Waqas Rana. Writing – review and editing: Hamza Ashraf, Zain Ali Nadeem, Khawaja Abdul Rehman, Shanzay Akhtar, Haider Ashfaq, Eeshal Fatima, Muhammad Sohaib Khan, Mahad Butt, Ibrahim Nagmeldin, and Waqas Rana, Aalaa Saleh, Hritvik Jain, and Raheel Ahmed.

## Disclosure

The authors have nothing to report.

## Conflicts of Interest

The authors declare no conflicts of interest.

## Supporting information

Supplementary Material.

## Data Availability

The data that support the findings of this study are available in the Supporting Information of this article.

## References

[1] T. J. Cahill and B. D. Prendergast , “Infective Endocarditis,” Lancet 387, no. 10021 (2016): 882–893. 26341945 10.1016/S0140-6736(15)00067-7

[2] J. Ambrosioni , M. Hernandez‐Meneses , A. Téllez , et al., “Infective Endocarditis: An Epidemiological Comparison Between the Pre‐ and Post‐*Staphylococcus aureus* eras,” International Journal of Cardiology 228 (2017): 1011–1016.

[3] W. Wilson , K. A. Taubert , M. Gewitz , et al., “Prevention of Infective Endocarditis: Guidelines From the American Heart Association,” Circulation 116, no. 15 (2007): 1736–1754. 17446442 10.1161/CIRCULATIONAHA.106.183095

[4] L. M. Baddour , W. R. Wilson , A. S. Bayer , et al., “Infective Endocarditis in Adults: Diagnosis, Antimicrobial Therapy, and Management of Complications: A Scientific Statement for Healthcare Professionals From the American Heart Association,” Circulation 132, no. 15 (2015): 1435–1486. 26373316 10.1161/CIR.0000000000000296

[5] N. Fernández‐Hidalgo , B. Almirante , P. Tornos , et al., “Contemporary Epidemiology and Prognosis of Healthcare‐Associated Infective Endocarditis,” Clinical Infectious Diseases 47, no. 10 (2008): 1287–1297. 18834314 10.1086/592576

[6] R. A. Seaton and D. A. Barr , “Outpatient Parenteral Antibiotic Therapy: Principles and Practice,” European Journal of Clinical Microbiology and Infectious Diseases 32, no. 1 (2013): 145–158. 10.1016/j.ejim.2013.03.014 23602223

[7] M. Twiddy , C. J. Czoski Murray , S. J. Mason , et al., “A Qualitative Study of Patients' Feedback About Outpatient Parenteral Antimicrobial Therapy (OPAT) Services in Northern England: Implications for Service Improvement,” BMJ Open 8, no. 1 (2018): 019099. 10.1136/bmjopen-2017-019099 PMC5781150 29326190

[8] M. Gilchrist and R. A. Seaton , “Outpatient Parenteral Antimicrobial Therapy (OPAT) in the UK: A Cross‐Sectional Survey of Health Professionals,” Journal of Antimicrobial Chemotherapy 70, no. 10 (2015): 2935–2942.

[9] A. D. Tice , S. J. Rehm , J. R. Dalovisio , et al., “Practice Guidelines for Outpatient Parenteral Antimicrobial Therapy,” Clinical Infectious Diseases 38, no. 12 (2004): 1651–1671. 15227610 10.1086/420939

[10] L. Bernard , P. El‐Hajj , B. Pron , et al., “Outpatient Parenteral Antimicrobial Therapy (OPAT) in France: Evaluation of Efficacy, Tolerability, and Cost,” Journal of Antimicrobial Chemotherapy 47, no. 1 (2001): 43–50.11152430

[11] W. Wen , H. Li , C. Wang , et al., “Efficacy and Safety of Outpatient Parenteral Antibiotic Therapy in Patients With Infective Endocarditis: A Meta‐Analysis,” Revista Española de Quimioterapia 35, no. 4 (2022): 370–377. 35652306 10.37201/req/011.2022 PMC9333124

[12] Cochrane Handbook for Systematic Reviews of Interventions, Cochrane Training n.d., accessed August 25, 2024, https://training.cochrane.org/handbook.

[13] B. Hutton , G. Salanti , D. M. Caldwell , et al., “The PRISMA Extension Statement for Reporting of Systematic Reviews Incorporating Network Meta‐Aanalyses of Health Care Interventions: Checklist and Explanations,” Annals of Internal Medicine 162 (2015): 777–784, 10.7326/M14-2385. 26030634

[14] J. A. Sterne , M. A. Hernán , B. C. Reeves , et al., “ROBINS‐I: A Tool for Assessing Risk of Bias in Non‐Randomised Studies of Interventions,” BMJ 355 (2016): i4919, Published 2016 Oct 12. 10.1136/bmj.i4919. 27733354 PMC5062054

[15] M. F. Freeman and J. W. Tukey , “Transformations Related to the Angular and the Square Root,” Annals of Mathematical Statistics 21 (1950): 607–611.

[16] J. IntHout , J. P. Ioannidis , and G. F. Borm , “The Hartung‐Knapp‐Sidik‐Jonkman Method for Random Effects Meta‐Analysis Is Straightforward and Considerably Outperforms the Standard DerSimonian‐Laird Method,” BMC Medical Research Methodology 14 (2014): 25, 10.1186/1471-2288-14-25. 24548571 PMC4015721

[17] J. J. Deeks , J. P. Higgins , and D. G. Altman , Cochrane Statistical Methods Group ., “Analysing Data and Undertaking Meta‐Analyses,” Cochrane Handbook for Systematic Reviews of Interventions (2019): 241–284, 10.1002/9781119536604.ch10.

[18] M. Egger , G. D. Smith , M. Schneider , and C. Minder , “Bias in Meta‐Analysis Detected by a Simple, Graphical Test,” BMJ 315 (1997): 629–634, 10.1136/BMJ.315.7109.629. 9310563 PMC2127453

[19] X. Kortajarena , M. A. Goenaga , M. Ibarguren , et al., “Outpatient Parenteral Antimicrobial Therapy for Infective Endocarditis in Patients Over 80 Years,” Revista espanola de quimioterapia: publicacion oficial de la Sociedad Espanola de Quimioterapia 30, no. 4 (2017): 276–279. 28585797

[20] J. M. Pericà s , J. Llopis , V. González‐Ramallo , et al., “Outpatient Parenteral Antibiotic Treatment for Infective Endocarditis: A Prospective Cohort Study From the GAMES Cohort,” Clinical Infectious Diseases 69, no. 10 (2019): 1690–1700, 10.1093/cid/ciz030. 30649282

[21] O. C. Durojaiye , R. Morgan , N. Chelaghma , and E. I. Kritsotakis , “Clinical Predictors of Outcome in Patients With Infective Endocarditis Receiving Outpatient Parenteral Antibiotic Therapy (OPAT),” Journal of Infection 83, no. 6 (2021): 644–649, 10.1016/j.jinf.2021.09.021. 34614400

[22] A. K. F. Htin , N. D. Friedman , A. Hughes , et al., “Outpatient Parenteral Antimicrobial Therapy Is Safe and Effective for the Treatment of Infective Endocarditis: A Retrospective Cohort Study,” Internal Medicine Journal 43, no. 6 (2013): 700–705, 10.1111/imj.12081. 23347167

[23] M. Pajarón , M. Lisa , M. F. Fernández‐Miera , et al., “Efficiency of a Self‐Administered Outpatient Parenteral Antimicrobial Therapy (s‐opat) for Infective Endocarditis Within the Context of a Shortened Hospital Admission Based on Hospital at Home Program,” Hospital Practice 45, no. 5 (2017): 246–252, 10.1080/21548331.2017.1398588. 29090606

[24] D. G. Partridge , E. O'Brien , and A. L. N. Chapman , “Outpatient Parenteral Antibiotic Therapy for Infective Endocarditis: A Review of 4 Years' Experience at a UK Centre,” Postgraduate Medical Journal 88, no. 1041 (2012): 377–381, 10.1136/postgradmedj-2011-130355. 22366395

[25] C. Cervera , A. del Río , L. García , et al., “Efficacy and Safety of Outpatient Parenteral Antibiotic Therapy for Infective Endocarditis: A Ten‐Year Prospective Study,” Enfermedades infecciosas y microbiología clínica 29, no. 8 (2011): 587–592, 10.1016/j.eimc.2011.05.007. 21723004

[26] A. Lacroix , M. Revest , S. Patrat‐Delon , et al., “Outpatient Parenteral Antimicrobial Therapy for Infective Endocarditis: A Cost‐Effective Strategy,” Médecine et maladies infectieuses 44, no. 7 (2014): 327–330, 10.1016/j.medmal.2014.05.001. 25022891

[27] M. R. Amodeo , T. Clulow , J. Lainchbury , et al., “Outpatient Intravenous Treatment for Infective Endocarditis: Safety, Effectiveness and One‐Year Outcomes,” Journal of Infection 59, no. 6 (2009): 387–393, 10.1016/j.jinf.2009.09.009. 19766136

[28] N. Vallejo Camazon , L. Mateu , G. Cediel , et al., “Long‐Term Antibiotic Therapy in Patients With Surgery‐Indicated Not Undergoing Surgery Infective Endocarditis,” Cardiology Journal 28, no. 4 (2021): 566–578, 10.5603/CJ.a2021.0054. 34031866 PMC8276997

[29] P. O. Campbell , K. Gallagher , S. C. Dalton , S. C. L. Metcalf , N. M. Douglas , and S. T. Chambers , “Safety and Clinical Outcomes of Outpatient Parenteral Antibiotic Therapy for Infective Endocarditis in Christchurch, New Zealand: A Retrospective Cohort Study,” International Journal of Infectious Diseases 134 (2023): 172–176, 10.1016/j.ijid.2023.06.008. 37331565

[30] S. Douiyeb , J. R. de la Court , B. Tuinte , et al., “Risk Factors for Readmission Among Patients Receiving Outpatient Parenteral Antimicrobial Therapy: A Retrospective Cohort Study,” International Journal of Clinical Pharmacy 44, no. 2 (2022): 557–563, 10.1007/s11096-022-01379-7. 35157228 PMC9007809

[31] S. Freling , N. Wald‐Dickler , J. Banerjee , et al., “Real‐World Application of Oral Therapy for Infective Endocarditis: A Multicenter, Retrospective, Cohort Study,” Clinical Infectious Diseases 77, no. 5 (2023): 672–679, 10.1093/cid/ciad119. 36881940

[32] R. García‐Carretero , O. Vazquez‐Gomez , B. Rodriguez‐Maya , G. Naranjo‐Mansilla , and E. Luna‐Heredia , “Infective Endocarditis in a Hospital‐at‐Home Setting: A Retrospective Analysis in a Peripheral Spanish Hospital,” Home Health Care Management & Practice 33 (2021): 108482232098851, 10.1177/1084822320988513.

[33] Y. Hamad , K. B. Nickel , M. A. Olsen , and I. A. George , “Outcomes of Ceftriaxone Compared With Cefazolin or Nafcillin/Oxacillin for Outpatient Therapy for Methicillin‐Sensitive *Staphylococcus aureus* Bloodstream Infections: Results From a Large United States Claims Database,” Open Forum Infectious Diseases 11, no. 2 (2024): ofad662, 10.1093/ofid/ofad662. 38352150 PMC10863560

[34] L. Herrera‐Hidalgo , J. M. Lomas‐Cabezas , L. E. López‐Cortés , et al., “Ampicillin Plus Ceftriaxone Combined Therapy for Enterococcus faecalis Infective Endocarditis in OPAT,” Journal of Clinical Medicine 11, no. 1 (2021): 7, 10.3390/jcm11010007. 35011748 PMC8745305

[35] P. R. Ingram , J. Ng , C. Mathieson , et al., “A Clinical and in Vitro Assessment of Outpatient Parenteral Benzylpenicillin and Ceftriaxone Combination Therapy for Enterococcal Endovascular Infections,” JAC‐Antimicrobial Resistance 3, no. 3 (2021): dlab128, 10.1093/jacamr/dlab128. 34377984 PMC8346702

[36] C. S. Kwok , J. J. Whittaker , C. Malbon , et al., “Outpatient Parenteral Antimicrobial Therapy (OPAT) Service Is Associated With Inpatient‐Bed Cost Savings,” British Journal of Cardiology 28, no. 3 (2021): 38, 10.5837/bjc.2021.038. 35747699 PMC8988795

[37] D. R. Schwiebert , D. S. Atanze , D. U. Iroegbu , D. M. Wilkins , and D. J. A. T. Sandoe , “Outpatient Parenteral Antibiotic Treatment for Infective Endocarditis: A Retrospective Observational Evaluation,” Clinical Medicine 24, no. 3 (2024): 100213, 10.1016/j.clinme.2024.100213. 38643831 PMC11101910

[38] J. Larioza , L. Heung , A. Girard , and R. B. Brown , “Management of Infective Endocarditis in Outpatients: Clinical Experience With Outpatient Parenteral Antibiotic Therapy,” Southern Medical Journal 102, no. 6 (2009): 575–579, 10.1097/SMJ.0b013e3181a4eef2. 19434034

[39] G. Lopardo , “Management of Endocarditis: Outpatient Parenteral Antibiotic Treatment in Argentina,” Chemotherapy 47, no. Suppl 1 (2001): 24–32, 10.1159/000048565. 11096186

[40] E. Aparicio‐Minguijón , J. Boán , A. Terrón , et al., “Dalbavancin as Sequential Therapy in Infective Endocarditis: Real‐Life Experience in Elder and Comorbid Patients,” Enfermedades Infecciosas y microbiología clínica 43, no. 2 (2025): 86–92, 10.1016/j.eimce.2024.04.012. 38902152

[41] M. V. Gil‐Navarro , L. E. Lopez‐Cortes , R. Luque‐Marquez , J. Galvez‐Acebal , and A. Alarcon‐Gonzalez , “Outpatient Parenteral Antimicrobial Therapy in Enterococcus faecalis Infective Endocarditis,” Journal of Clinical Pharmacy and Therapeutics 43, no. 2 (2018): 220–223, 10.1111/jcpt.12635. 29030859

[42] M. Suárez , A. Pérez‐Landeiro , A. Sanjurjo , et al., “Comparison of Dalbavancin With Standard of Care in the Management of Infective Endocarditis: Efficacy, Safety, and Cost Analysis,” International Journal of Infectious Diseases 138 (2024): 41–45, 10.1016/j.ijid.2023.11.003. 37931892

[43] M. Gilchrist , D. Barr , F. Drummond , et al., “Outpatient Parenteral Antimicrobial Therapy (OPAT) in the UK: Findings From the BSAC National Outcomes Registry (2015‐19),” Journal of Antimicrobial Chemotherapy 77, no. 5 (2022): 1481–1490, 10.1093/jac/dkac047. 35187565

[44] S. A. Mohammed , J. A. Roberts , M. O. Cotta , et al., “Safety and Efficacy of Outpatient Parenteral Antimicrobial Therapy: A Systematic Review and Meta‐Analysis of Randomized Clinical Trials,” International Journal of Antimicrobial Agents 64, no. 2 (2024): 107263, 10.1016/j.ijantimicag.2024.107263. 38960209

[45] G. Agnihotri , A. E. Gross , M. Seok , et al., “Decreased Hospital Readmissions After Programmatic Strengthening of an Outpatient Parenteral Antimicrobial Therapy (OPAT) Program,” Antimicrobial Stewardship & Healthcare Epidemiology: ASHE 3, no. 1 (2023): e33, 10.1017/ash.2022.330. 36865701 PMC9972539

[46] L. K. Certain , R. J. Benefield , M. Newman , M. Zhang , and F. O. Thomas , “A Quality Initiative to Improve Postdischarge Care for Patients on Outpatient Parenteral Antimicrobial Therapy,” Open Forum Infectious Diseases 9, no. 7 (2022): ofac199, 10.1093/ofid/ofac199. 35794930 PMC9251666

[47] Z. T. Wolie , J. A. Roberts , M. Gilchrist , K. McCarthy , and F. B. Sime , “Current Practices and Challenges of Outpatient Parenteral Antimicrobial Therapy: A Narrative Review,” Journal of Antimicrobial Chemotherapy 79, no. 9 (2024): 2083–2102, 10.1093/jac/dkae177. 38842523 PMC11368434

[48] Y. Sharma , M. Miller , B. Kaambwa , et al., “Factors Influencing Early and Late Readmissions in Australian Hospitalised Patients and Investigating Role of Admission Nutrition Status as a Predictor of Hospital Readmissions: A Cohort Study,” BMJ Open 8 (2018): e022246, 10.1136/bmjopen-2018-022246. PMC6020977 29950478

[49] R. A. Nishimura , C. M. Otto , R. O. Bonow , et al., “2014 AHA/ACC Guideline for the Management of Patients With Valvular Heart Disease,” Journal of Thoracic and Cardiovascular Surgery 148, no. 1 (2014): e1–e132, 10.1016/j.jtcvs.2014.05.014. 24939033

[50] V. Delgado , N. Ajmone Marsan , S. de Waha , et al., “2023 ESC Guidelines for the management of endocarditis: Developed by the Task Force on the Management of Endocarditis of the European Society of Cardiology (ESC) Endorsed by the European Association for Cardio-Thoracic Surgery (EACTS) and the European Association of Nuclear Medicine (EANM),” European Heart Journal 44, no. 39 (2023): 3948–4042, 10.1093/eurheartj/ehad193.

[51] J. Halilovic , C. L. Christensen , and H. H. Nguyen , “Managing an Outpatient Parenteral Antibiotic Therapy Team: Challenges and Solutions,” Therapeutics and Clinical Risk Management 10 (2014): 459–465, 10.2147/TCRM.S48906. 24971015 PMC4069209

[52] S. C. Keller , K. Dzintars , L. A. Gorski , D. Williams , and S. E. Cosgrove , “Antimicrobial Agents and Catheter Complications in Outpatient Parenteral Antimicrobial Therapy,” Pharmacotherapy: The Journal of Human Pharmacology and Drug Therapy 38, no. 4 (2018): 476–481, 10.1002/phar.2099. PMC5902416 29493791

[53] C. M. Kaul , M. Haller , J. Yang , et al., “Assessment of Risk Factors Associated With Outpatient Parenteral Antimicrobial Therapy (OPAT) Complications: A Retrospective Cohort Study,” Antimicrobial Stewardship & Healthcare Epidemiology 2, no. 1 (2022): e183, 10.1017/ash.2022.313. 36406163 PMC9672913

[54] J. Suzuki , J. Johnson , M. Montgomery , M. Hayden , and C. Price , “Outpatient Parenteral Antimicrobial Therapy Among People Who Inject Drugs: A Review of the Literature,” Open Forum Infectious Diseases 5, no. 9 (2018): ofy194, 10.1093/ofid/ofy194. 30211247 PMC6127783

[55] A. Appa , C. Marquez , and V. Jain , “Home‐Based Outpatient Parenteral Antibiotic Therapy at an Urban Safety Net Hospital: Comparing Outcomes in Persons With and Without Noninjection Drug Use,” Open Forum Infectious Diseases 7, no. 5 (2020): ofaa162, 10.1093/ofid/ofaa162. 32494584 PMC7252283

[56] A. M. Beieler , T. H. Dellit , J. D. Chan , et al., “Successful Iimplementation of Outpatient Parenteral Antimicrobial Therapy at a Medical Respite Facility for Homeless Patients,” Journal of Hospital Medicine 11, no. 8 (2016): 531–535, 10.1002/jhm.2597. 27120700

[57] J. Ho , S. Archuleta , Z. Sulaiman , and D. Fisher , “Safe and Successful Treatment of Intravenous Drug Users With a Peripherally Inserted Central Catheter in an Outpatient Parenteral Antibiotic Treatment Service,” Journal of Antimicrobial Chemotherapy 65, no. 12 (2010): 2641–2644, 10.1093/jac/dkq355. 20864497

[58] Y. Haddadin , P. Annamaraju , and H. Regunath , “Central Line–Associated Blood Stream Infections. 2022 Nov 26.” StatPearls [Internet] (Treasure Island (FL): StatPearls Publishing, 2025. 28613641

[59] B. Hoen , “Epidemiology and Antibiotic Treatment of Infective Endocarditis: An Update,” Heart 92, no. 11 (2006): 1694–1700, 10.1136/hrt.2005.072595. 17041124 PMC1861255

[60] A. Gagneux‐Brunon , A. Pouvaret , N. Maillard , et al., “Acute Kidney Injury in Infective Endocarditis: A Retrospective Analysis,” Médecine et maladies infectieuses 49, no. 7 (2019): 527–533, 10.1016/j.medmal.2019.03.015. 30955847

[61] P. Shakeraneh , T. Fazili , D. Wang , et al., “Nephrotoxicity Risk and Clinical Effectiveness of Continuous Versus Intermittent Infusion Vancomycin Among Patients in an Outpatient Parenteral Antimicrobial Therapy Program,” Pharmacotherapy: The Journal of Human Pharmacology and Drug Therapy 40, no. 4 (2020): 357–362, 10.1002/phar.2381. 32090347

[62] L. Østergaard , M. Voldstedlund , N. E. Bruun , et al., “Recurrence of Bacteremia and Infective Endocarditis According to Bacterial Species of Index Endocarditis Episode,” Infection 51, no. 6 (2023): 1739–1747, 10.1007/s15010-023-02068-x. 37395924 PMC10665237

[63] W. Hu , X. Wang , and G. Su , “Infective Endocarditis Complicated by Embolic Events: Pathogenesis and Predictors,” Clinical Cardiology 44, no. 3 (2021): 307–315, 10.1002/clc.23554. 33527443 PMC7943911

