# Supplementary material for: Safety and Efficacy of Outpatient Parenteral Antibiotic Therapy (OPAT) in Patients With Infective Endocarditis: A Systematic Review and Meta‐Analysis
Source: Clin Cardiol. 2025 May 14;48(5):e70147. doi: 10.1002/clc.70147 (PMC12076119; doi:10.1002/clc.70147)
Supplement: Supplementary file 1 — Supplementary Material. [file CLC-48-e70147-s001.docx]

**Supplementary File**

**Safety and Efficacy of Outpatient Parenteral Antibiotic Therapy (OPAT) in Patients with Infective Endocarditis: A Systematic Review and Meta-Analysis**

Hamza Ashraf, Zain Ali Nadeem, Khawaja Abdul Rehman, Shanzay Akhtar, Haider Ashfaq, Muhammad Sohaib Khan, Mahad Butt, Ibrahim Nagmeldin, Eeshal Fatima, Waqas Rana, Aalaa Saleh, Hritvik Jain and Raheel Ahmed

**Clinical Cardiology**

This supplemental material is provided by the authors to help them better understand their work.

**Legend

Supplementary Table 1.** PRISMA checklist for the systematic review and meta-analysis.

**Supplementary Table 2.** Search strategy and databases used for literature selection.

**Supplementary Table 3**. Risk of bias assessment for included studies using the ROBINS-I tool.

**Supplementary Figure 1.** Traffic light plot (A) and summary plot (B) presenting the quality assessment of included studies using the risk of bias visualization (Robvis) tool

**Supplementary Figure 2.** Sensitivity analysis for mortality rate during the treatment period.

**Supplementary Figure 3**. Sensitivity analysis for relapse rate during the treatment period

**Supplementary Figure 4.** Sensitivity analysis for readmission rate during the treatment period.

**Supplementary Figure 5.** Sensitivity analysis for the percentage of patients requiring valve replacement or cardiac surgery during the treatment period.

**Supplementary Figure 6.** Sensitivity analysis for mortality rate during the follow-up period.

**Supplementary Figure 7.** Sensitivity analysis for relapse rate during the follow-up period

**Supplementary Figure 8**. Sensitivity analysis for readmission rate during the follow-up period.

**Supplementary Figure 9.** Sensitivity analysis for the percentage of patients requiring valve replacement or cardiac surgery during the follow-up period.

**Supplementary Figure 10.** Forest plot showing the incidence of drug allergy or complications in patients treated with OPAT.

**Supplementary Figure 11**. Forest plot showing the incidence of acute kidney injury or failure in patients treated with OPAT.

**Supplementary Figure 12**. Forest plot showing the incidence of IV-line-related adverse events in patients treated with OPAT.

**Supplementary Figure 13.** Forest plot showing the incidence of *Clostridioides difficile* colitis in patients treated with OPAT.

**Supplementary Figure 14.** Forest plot showing the incidence of PICC line complications in patients treated with OPAT.

**Supplementary Figure 15.** Forest plot showing the incidence of valvular regurgitation in patients treated with OPAT.

**Supplementary Figure 16.** Forest plot showing the incidence of heart failure in patients treated with OPAT.

**Supplementary Figure 17.** Forest plot showing the incidence of arrhythmia in patients treated with OPAT.

**Supplementary Figure 18**. Forest plot showing the incidence of stroke in patients treated with OPAT.

**Supplementary Figure 19.** Forest plot showing the incidence of persistent bacteremia in patients treated with OPAT.

**Supplementary Figure 20.** Forest plot showing the incidence of sepsis in patients treated with OPAT.

**Supplementary Figure 21**. Forest plot showing the incidence of embolic complications in patients treated with OPAT.

**Supplementary Figure 22**. Sensitivity analysis for drug allergy or complication.

**Supplementary Figure 23**. Sensitivity analysis for acute kidney injury or failure.

**Supplementary Figure 24**. Sensitivity analysis for IV-line related adverse events.

**Supplementary Figure 25**. Sensitivity analysis for *Clostridioides difficile* colitis.

**Supplementary Figure 26**. Sensitivity analysis for PICC line complications.

**Supplementary Figure 27**. Sensitivity analysis for valvular regurgitation.

**Supplementary Figure 28**. Sensitivity analysis for heart failure.

**Supplementary Figure 29**. Sensitivity analysis for arrhythmia.

**Supplementary Figure 30.** Sensitivity analysis for stroke.

**Supplementary Figure 31**. Sensitivity analysis for persistent bacteremia.

**Supplementary Figure 32**. Sensitivity analysis for sepsis.

**Supplementary Figure 33**. Sensitivity analysis for embolic complications.

**Supplementary Figure 34**. Funnel plot for publication bias in patients with heart failure.

**Supplementary Figure 35**. Funnel plot for publication bias in mortality rate during the treatment period.

**Supplementary Figure 36**. Funnel plot for publication bias in readmission rate during the treatment period.

**Supplementary Figure 37**. Funnel plot for publication bias in mortality rate during the follow-up period

**Supplementary Figure 38**. Funnel plot for publication bias in relapse rate during the follow-up period.

**Supplementary Figure 39**. Funnel plot for publication bias in readmission rate during the follow-up period.

**Supplementary Figure 40**. Funnel plot for publication bias in patients requiring valve replacement or cardiac surgery during the follow-up period.

**Supplementary Figure 41**. Funnel plot for publication bias in patients with a drug allergy or complication.

**Supplementary Table 1.** PRISMA checklist for the systematic review and meta-analysis.

| **Section and Topic** | **Item #** | **Checklist item** | **Location where item is reported** |
| --- | --- | --- | --- |
| **TITLE** | | |  |
| Title | 1 | Identify the report as a systematic review. | Page 1 |
| **ABSTRACT** | | |  |
| Abstract | 2 | See the PRISMA 2020 for Abstracts checklist. | Page 3 |
| **INTRODUCTION** | | |  |
| Rationale | 3 | Describe the rationale for the review in the context of existing knowledge. | Page 4 |
| Objectives | 4 | Provide an explicit statement of the objective(s) or question(s) the review addresses. | Page 4 |
| **METHODS** | | |  |
| Eligibility criteria | 5 | Specify the inclusion and exclusion criteria for the review and how studies were grouped for the syntheses. | Page 4 |
| Information sources | 6 | Specify all databases, registers, websites, organisations, reference lists and other sources searched or consulted to identify studies. Specify the date when each source was last searched or consulted. | Page 5 |
| Search strategy | 7 | Present the full search strategies for all databases, registers and websites, including any filters and limits used. | 5, Supplementary Material |
| Selection process | 8 | Specify the methods used to decide whether a study met the inclusion criteria of the review, including how many reviewers screened each record and each report retrieved, whether they worked independently, and if applicable, details of automation tools used in the process. | Page 5 |
| Data collection process | 9 | Specify the methods used to collect data from reports, including how many reviewers collected data from each report, whether they worked independently, any processes for obtaining or confirming data from study investigators, and if applicable, details of automation tools used in the process. | N/A |
| Data items | 10a | List and define all outcomes for which data were sought. Specify whether all results that were compatible with each outcome domain in each study were sought (e.g. for all measures, time points, analyses), and if not, the methods used to decide which results to collect. | Page 5,6 |
|  | 10b | List and define all other variables for which data were sought (e.g. participant and intervention characteristics, funding sources). Describe any assumptions made about any missing or unclear information. | Page 5,6 |
| Study risk of bias assessment | 11 | Specify the methods used to assess risk of bias in the included studies, including details of the tool(s) used, how many reviewers assessed each study and whether they worked independently, and if applicable, details of automation tools used in the process. | Page 6 |
| Effect measures | 12 | Specify for each outcome the effect measure(s) (e.g. risk ratio, mean difference) used in the synthesis or presentation of results. | Page 6 |
| Synthesis methods | 13a | Describe the processes used to decide which studies were eligible for each synthesis (e.g. tabulating the study intervention characteristics and comparing against the planned groups for each synthesis (item #5)). | Page 6 |
|  | 13b | Describe any methods required to prepare the data for presentation or synthesis, such as handling of missing summary statistics, or data conversions. | Page 6 |
|  | 13c | Describe any methods used to tabulate or visually display results of individual studies and syntheses. | Page 6 |
|  | 13d | Describe any methods used to synthesize results and provide a rationale for the choice(s). If meta-analysis was performed, describe the model(s), method(s) to identify the presence and extent of statistical heterogeneity, and software package(s) used. | Page 6 |
|  | 13e | Describe any methods used to explore possible causes of heterogeneity among study results (e.g. subgroup analysis, meta-regression). | Page 6 |
|  | 13f | Describe any sensitivity analyses conducted to assess robustness of the synthesized results. | Page 6 |
| Reporting bias assessment | 14 | Describe any methods used to assess risk of bias due to missing results in a synthesis (arising from reporting biases). | Page 6 |
| Certainty assessment | 15 | Describe any methods used to assess certainty (or confidence) in the body of evidence for an outcome. | N/A |
| **RESULTS** | | |  |
| Study selection | 16a | Describe the results of the search and selection process, from the number of records identified in the search to the number of studies included in the review, ideally using a flow diagram. | Page 6,7 |
|  | 16b | Cite studies that might appear to meet the inclusion criteria, but which were excluded, and explain why they were excluded. | Page 7 |
| Study characteristics | 17 | Cite each included study and present its characteristics. | Page 6,7 |
| Risk of bias in studies | 18 | Present assessments of risk of bias for each included study. | Page 7 |
| Results of individual studies | 19 | For all outcomes, present, for each study: (a) summary statistics for each group (where appropriate) and (b) an effect estimate and its precision (e.g. confidence/credible interval), ideally using structured tables or plots. | Page 7,8 |
| Results of syntheses | 20a | For each synthesis, briefly summarise the characteristics and risk of bias among contributing studies. | Page 7 |
|  | 20b | Present results of all statistical syntheses conducted. If meta-analysis was done, present for each the summary estimate and its precision (e.g. confidence/credible interval) and measures of statistical heterogeneity. If comparing groups, describe the direction of the effect. | Page 6-8 |
|  | 20c | Present results of all investigations of possible causes of heterogeneity among study results. | Page 7-8 |
|  | 20d | Present results of all sensitivity analyses conducted to assess the robustness of the synthesized results. | Page 7-8 |
| Reporting biases | 21 | Present assessments of risk of bias due to missing results (arising from reporting biases) for each synthesis assessed. | Page 7-8 |
| Certainty of evidence | 22 | Present assessments of certainty (or confidence) in the body of evidence for each outcome assessed. | N/A |
| **DISCUSSION** | | |  |
| Discussion | 23a | Provide a general interpretation of the results in the context of other evidence. | Page 9 |
|  | 23b | Discuss any limitations of the evidence included in the review. | Page 11 |
|  | 23c | Discuss any limitations of the review processes used. | Page 11 |
|  | 23d | Discuss implications of the results for practice, policy, and future research. | Page 11 |
| **OTHER INFORMATION** | | |  |
| Registration and protocol | 24a | Provide registration information for the review, including register name and registration number, or state that the review was not registered. | Page 4 |
|  | 24b | Indicate where the review protocol can be accessed, or state that a protocol was not prepared. | Page 4 |
|  | 24c | Describe and explain any amendments to information provided at registration or in the protocol. | N/A |
| Support | 25 | Describe sources of financial or non-financial support for the review, and the role of the funders or sponsors in the review. | Page 11 |
| Competing interests | 26 | Declare any competing interests of review authors. | Page 11 |
| Availability of data, code and other materials | 27 | Report which of the following are publicly available and where they can be found: template data collection forms; data extracted from included studies; data used for all analyses; analytic code; any other materials used in the review. | N/A |

**Supplementary Table 2.** Search strategy and databases used for literature selection.

| **Database** | **Search Strategy** | **# of Results** |
| --- | --- | --- |
| PubMed | (((((((((("Endocarditis"[Mesh]) OR (Infective Endocarditides)) OR (Endocarditis, Infective)) OR (Endocarditides, Infective)) OR (Infective Endocarditis)) OR (Endocarditides)) OR ("Endocarditis, Bacterial"[Mesh])) OR (Bacterial Endocarditides)) OR (Bacterial Endocarditis)) OR (Endocarditides, Bacterial)) AND (((((("Infusions, Parenteral"[Mesh]) AND "Outpatients"[Mesh]) OR (Outpatient parenteral antibiotic)) OR (Outpatient parenteral antibiotic therapy)) OR (OPAT)) OR (Out-patient parenteral antibiotic)) | 130 |
| Scopus | ( TITLE-ABS-KEY ( “infective endocarditis” OR endocarditis ) AND TITLE-ABS-KEY ( "outpatient parenteral antibiotic therap*" OR opat OR "outpatient parenteral antibioti*" ) ) | 189 |
| Cochrane CENTRAL | *Set#1*  (infective endocarditis OR infective endocarditides)  *Set#2*  (Outpatient parenteral antibiotic therapy OR OPAT OR Outpatient parenteral antibiotic)  *Set#3*  #1 AND #2 | 17 |
| Google Scholar | "endocarditis" "infective endocarditis" "OPAT" OR "outpatient parenteral antibiotic therapy" OR "outpatient parenteral antibiotic" | 1480 |

**Supplementary Table 3**. Risk of bias assessment for included studies using the ROBINS-I tool.

| **Study Name** | **D1** | **D2** | **D3** | **D4** | **D5** | **D6** | **D7** | **Overall Bias** |
| --- | --- | --- | --- | --- | --- | --- | --- | --- |
| **Amodeo 2009** | Moderate | Moderate | Low | Low | Low | Low | Low | Moderate |
| **Cervera 2011** | Moderate | Moderate | Low | Low | Low | Serious | Low | Serious |
| **Durojaiye 2021** | Serious | Low | Low | Low | Low | Serious | Moderate | Serious |
| **Htin 2013** | Moderate | Low | Low | Moderate | Low | Low | Moderate | Moderate |
| **Kortajarena 2017** | Serious | Moderate | Low | Low | Low | Moderate | Low | Serious |
| **Lacroix 2017** | Serious | Low | Low | Moderate | Low | Moderate | Moderate | Serious |
| **Pajarón 2016** | Moderate | Low | Low | Moderate | Low | Moderate | Low | Moderate |
| **Partridge 2012** | Moderate | Moderate | Low | Low | Low | Moderate | Low | Moderate |
| **Pericàs 2019** | Moderate | Moderate | Low | Low | Moderate | Low | Moderate | Moderate |
| **Aparicio-Minguijón E 2024** | Moderate | Moderate | Low | Low | Moderate | Moderate | Moderate | Moderate |
| **Camazon NV 2021** | Moderate | Moderate | Low | Moderate | Moderate | Moderate | Moderate | Moderate |
| **Campbell PO 2023** | Moderate | Low | Low | Moderate | Low | Low | Low | Moderate |
| **Douiyeb S 2022** | Moderate | Low | Low | Moderate | Low | Moderate | Low | Moderate |
| **Freling S 2023** | Moderate | Low | Low | Moderate | Low | Moderate | Low | Moderate |
| **Garcia-Carretero R 2021** | Moderate | Low | Low | Moderate | Moderate | Moderate | Low | Moderate |
| **Gil-navarro 2017** | Moderate | Moderate | Low | Low | Low | Moderate | Low | Moderate |
| **Hamad Y 2023** | Moderate | Moderate | Low | Low | Moderate | Moderate | Low | Moderate |
| **Herrera-Hidalgo L 2021** | Serious | Moderate | Low | Moderate | Low | Moderate | Low | Serious |
| **Ingram PR 2021** | Serious | Moderate | Low | Moderate | Low | Moderate | Low | Moderate |
| **Kwok CS 2021** | Moderate | Moderate | Low | Moderate | Low | Moderate | Low | Moderate |
| **Iarioza 2009** | Moderate | Moderate | Low | Low | Moderate | Low | Low | Moderate |
| **Iopardo 2001** | Moderate | Moderate | Low | Low | Moderate | Moderate | Low | Moderate |
| **Pericás JM 2022** | Serious | Moderate | Low | Moderate | Low | Low | Moderate | Serious |
| **Schwiebert R 2023** | Serious | Moderate | Low | Moderate | Low | Low | Moderate | Serious |
| **Suárez M 2023** | Serious | Low | Low | Low | Moderate | Moderate | Serious | Serious |

**D1**= Bias due to confounding, **D2**= Bias in selection of participants into the study, **D3**= Bias in classification of interventions **D4**= Bias due to deviations from intended interventions, **D5**= Bias due to missing data, **D6**= Bias in measurement of outcomes **D7**= Bias in selection of the reported results


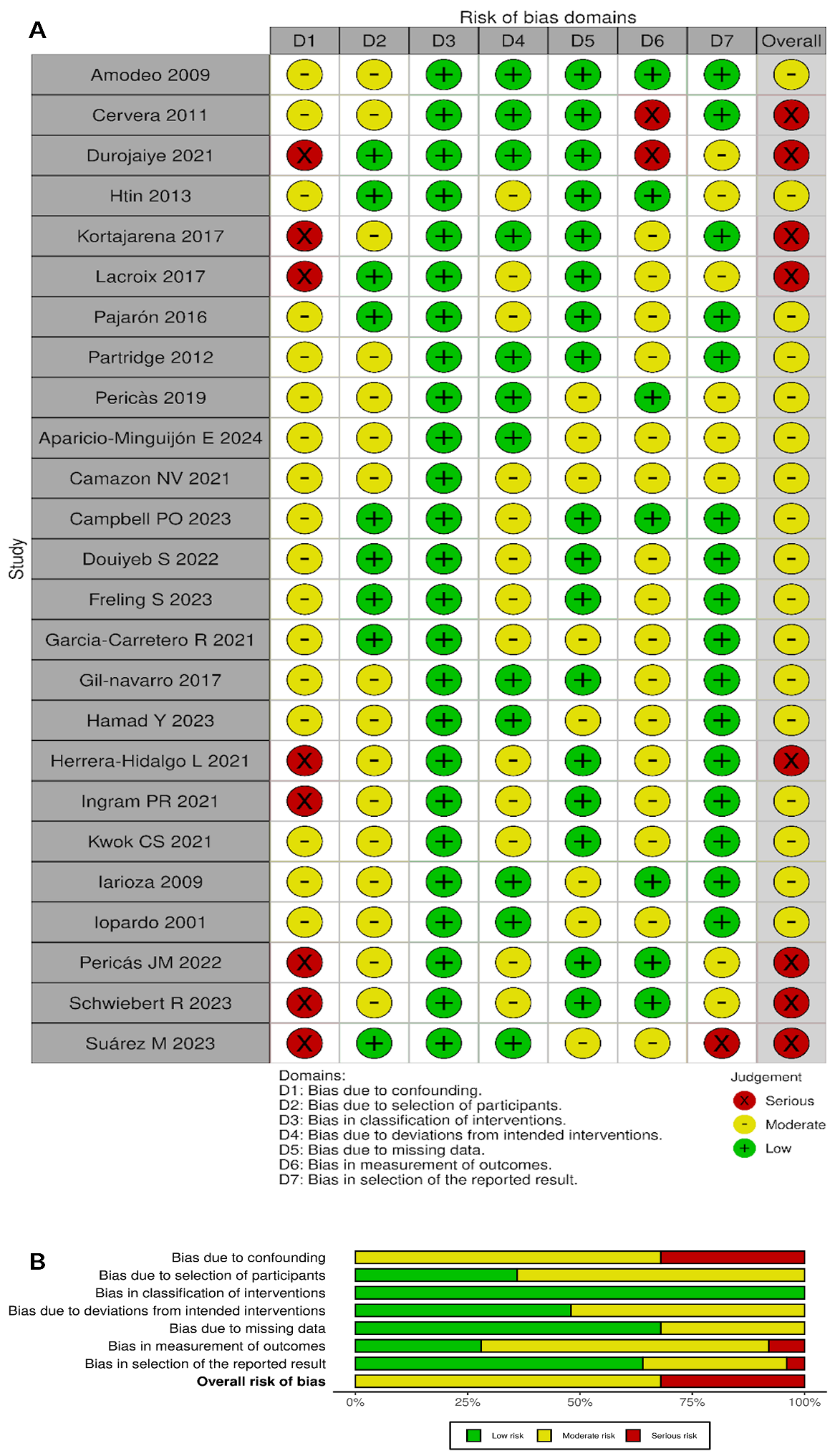


**Supplementary Figure 1.** Traffic light plot (A) and summary plot (B) presenting the quality assessment of included studies using the risk of bias visualization (Robvis) tool


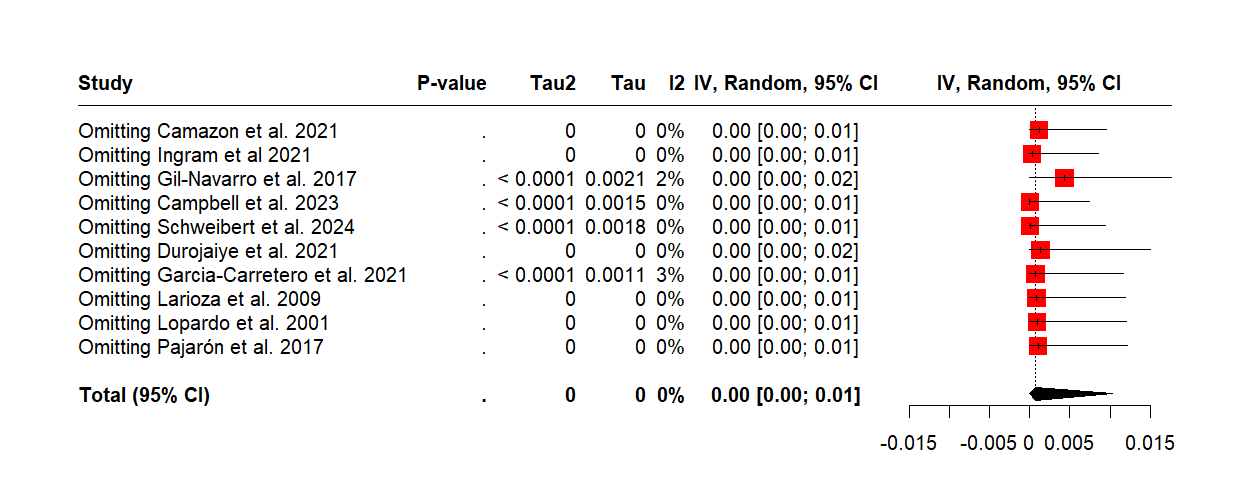


**Supplementary Figure 2.** Sensitivity analysis for mortality rate during the treatment period.


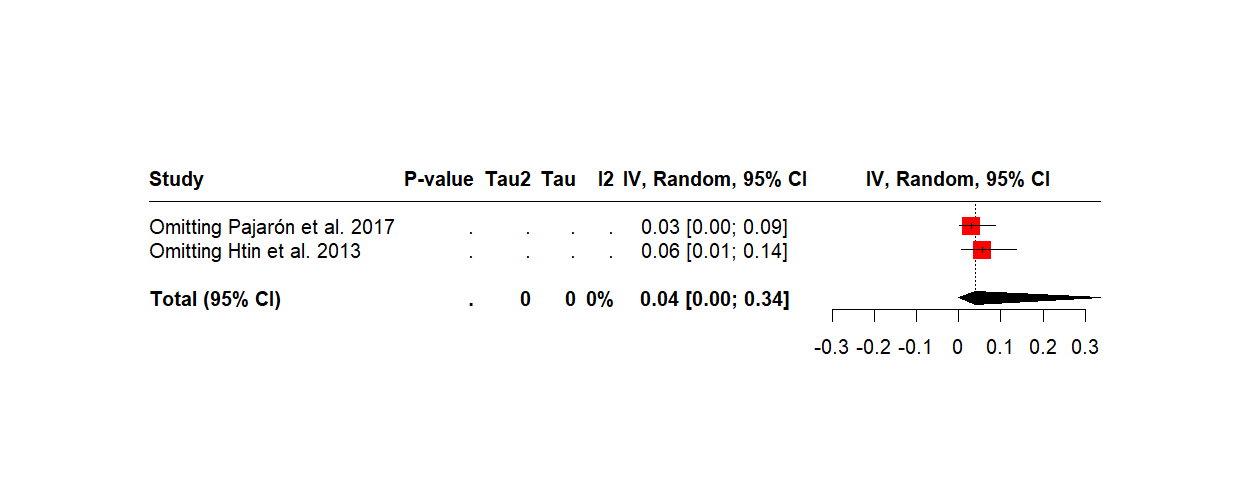


**Supplementary Figure 3**. Sensitivity analysis for relapse rate during the treatment period.


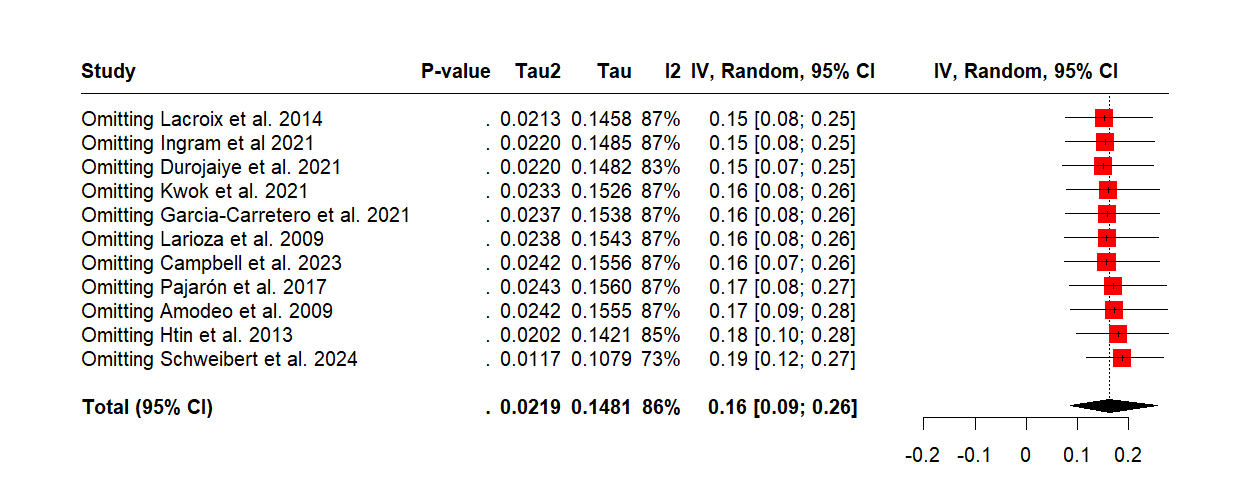
**Supplementary Figure 4.** Sensitivity analysis for readmission rate during the treatment period.


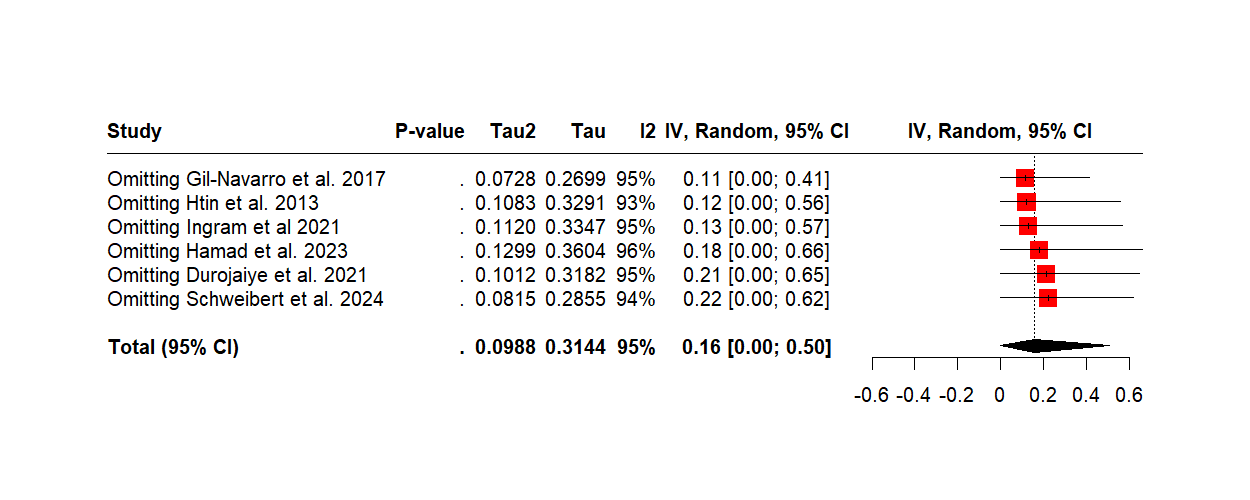


**Supplementary Figure 5.** Sensitivity analysis for the percentage of patients requiring valve replacement or cardiac surgery during the treatment period.

**Supplementary Figure 6.** Sensitivity analysis for mortality rate during the follow-up period.


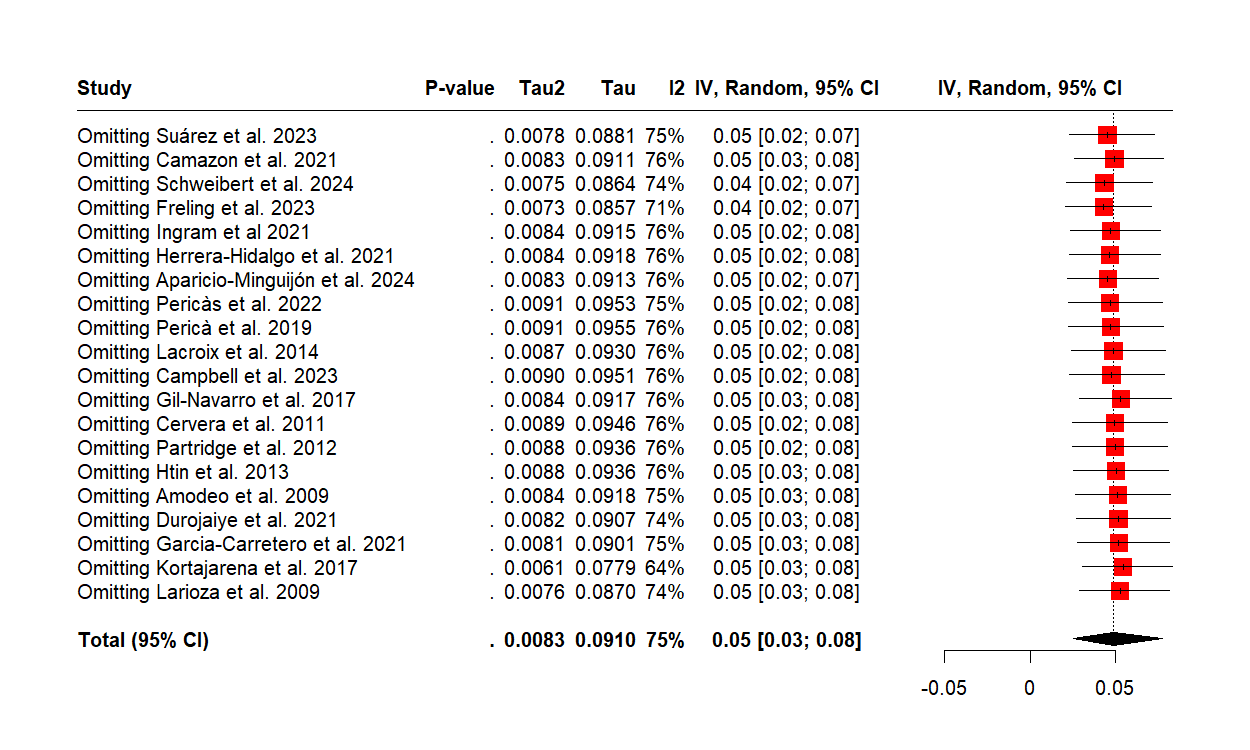


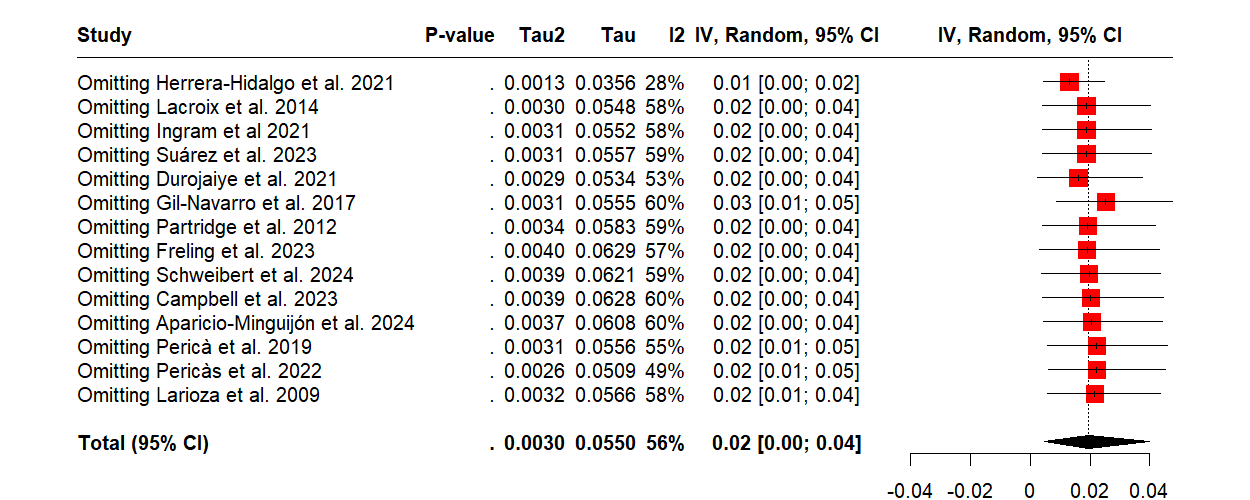


**Supplementary Figure 7:** Sensitivity analysis for relapse rate during the follow-up period

**Supplementary Figure 8**. Sensitivity analysis for readmission rate during the follow-up period.


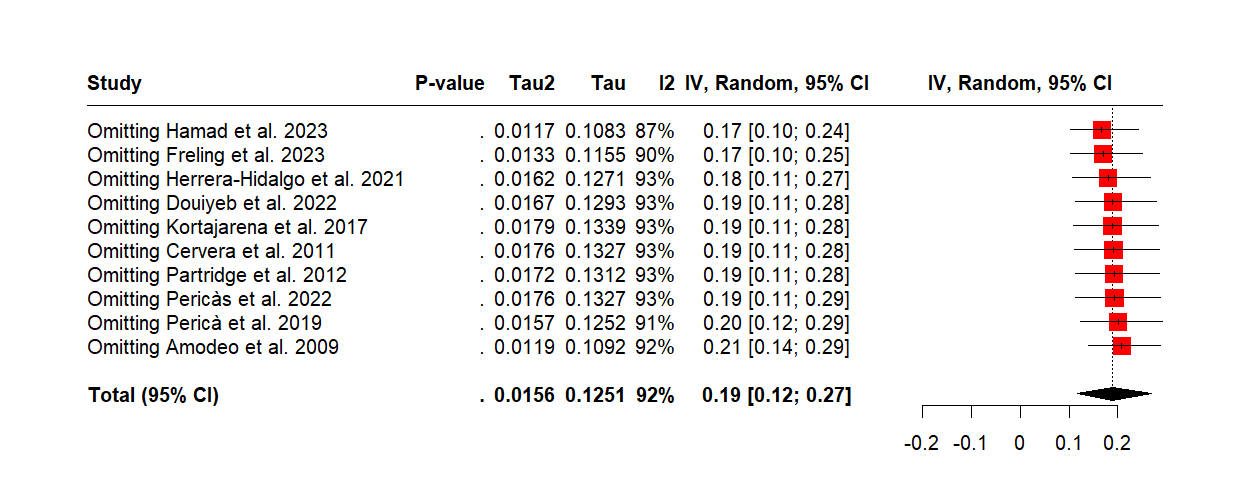


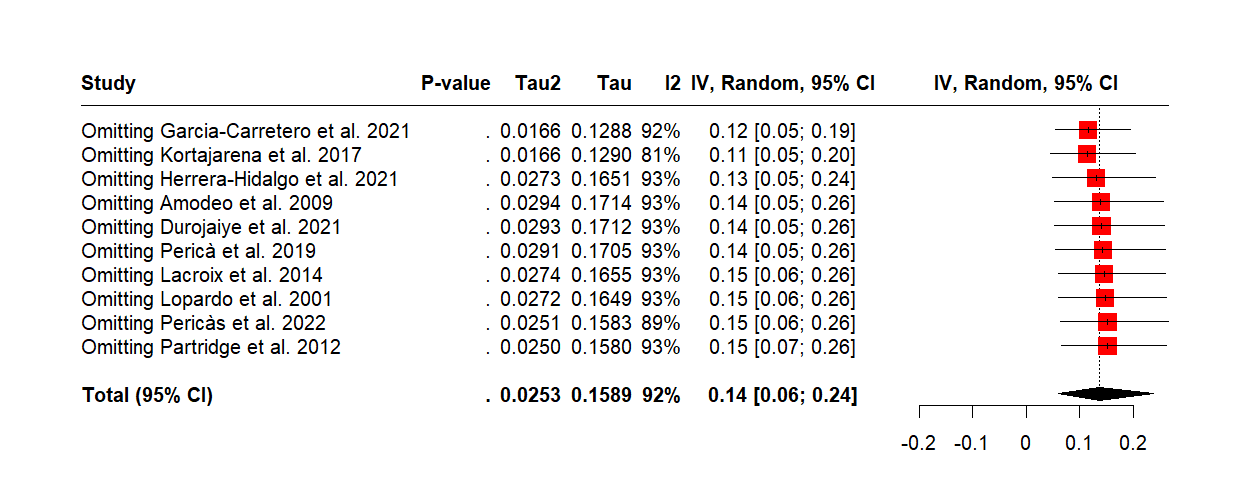


**Supplementary Figure 9.** Sensitivity analysis for the percentage of patients requiring valve replacement or cardiac surgery during the follow-up period.


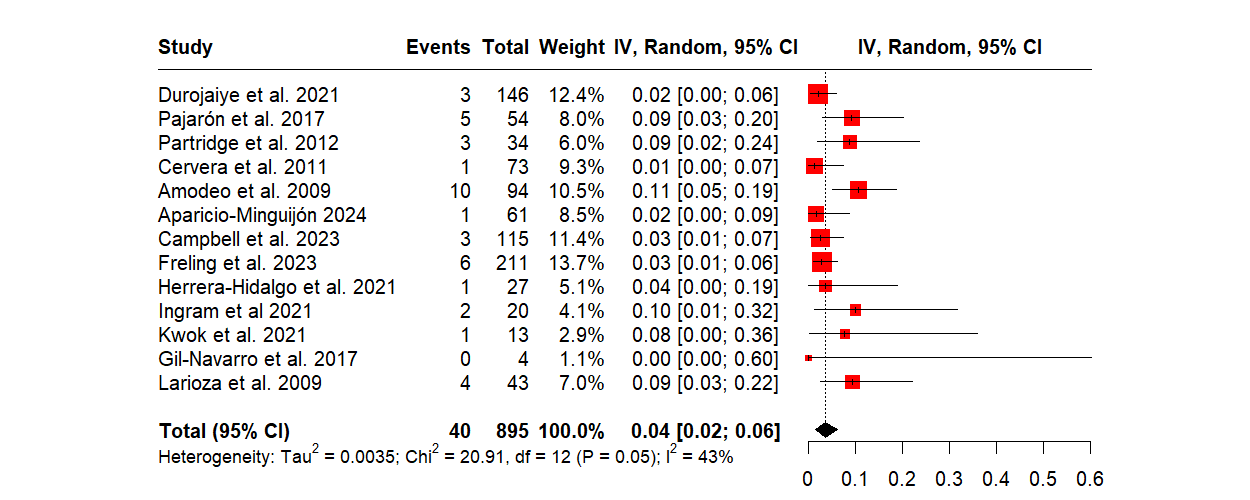


**Supplementary Figure 10**: Forest plot showing the incidence of drug allergy or complications in patients treated with OPAT.


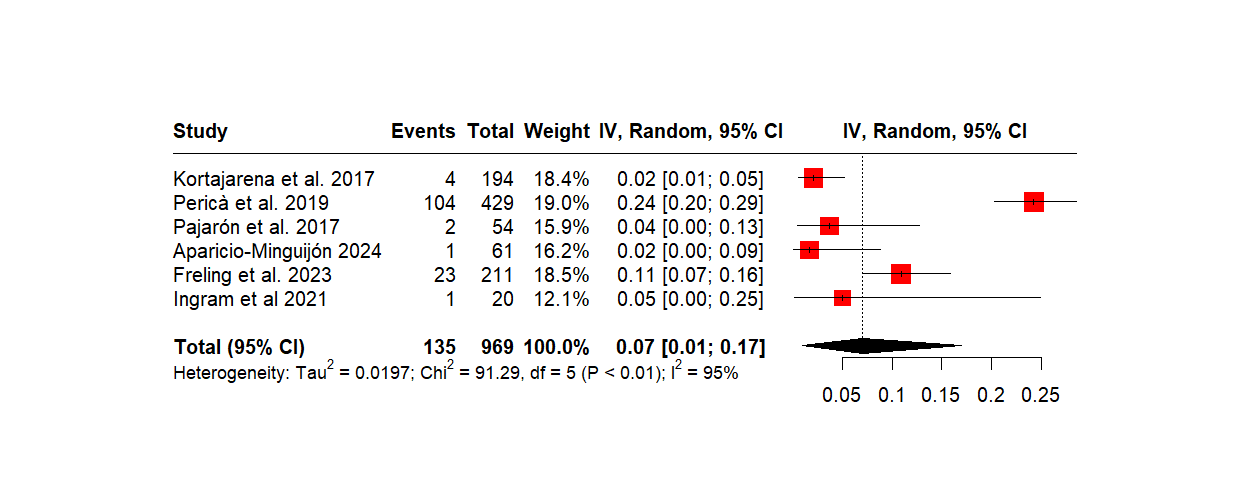


**Supplementary Figure 11**: Forest plot showing the incidence of acute kidney injury or failure in patients treated with OPAT.


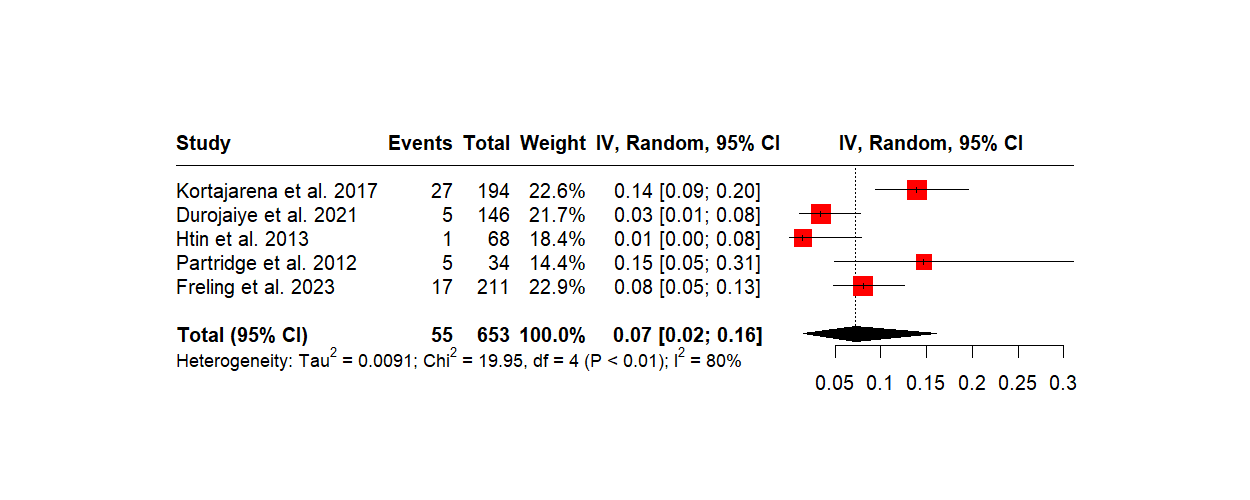


**Supplementary Figure 12**: Forest plot showing the incidence of IV-line-related adverse events in patients treated with OPAT.


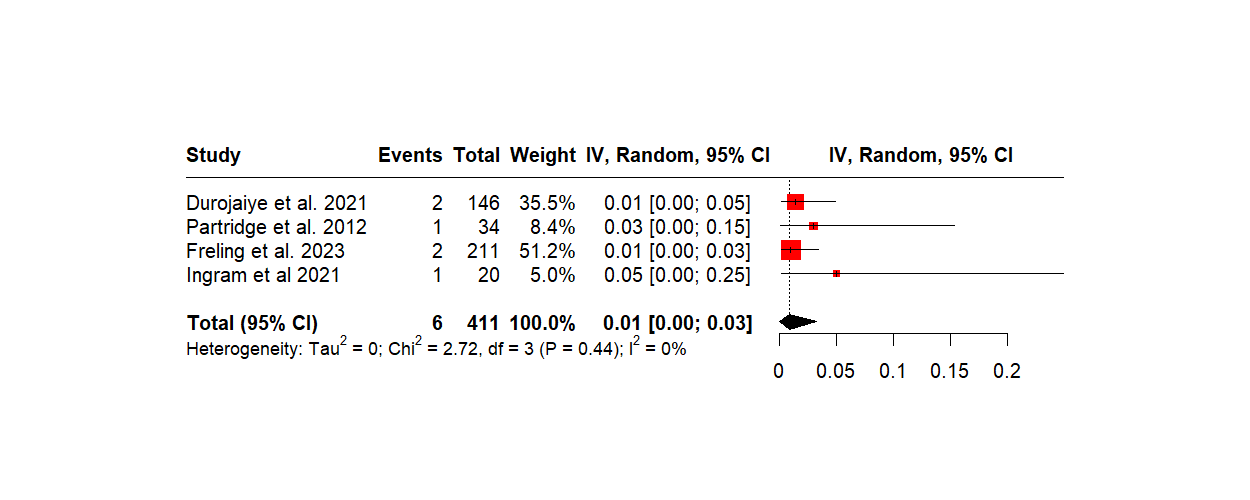


**Supplementary Figure 13:** Forest plot showing the incidence of *Clostridioides difficile* colitis in patients treated with OPAT.


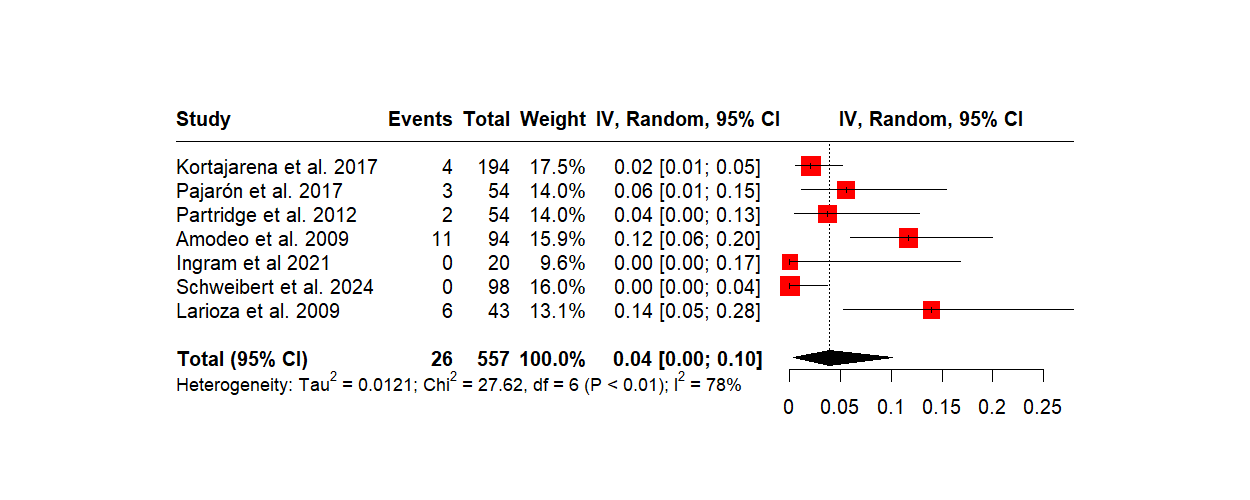


**Supplementary Figure 14:** Forest plot showing the incidence of PICC line complications in patients treated with OPAT.


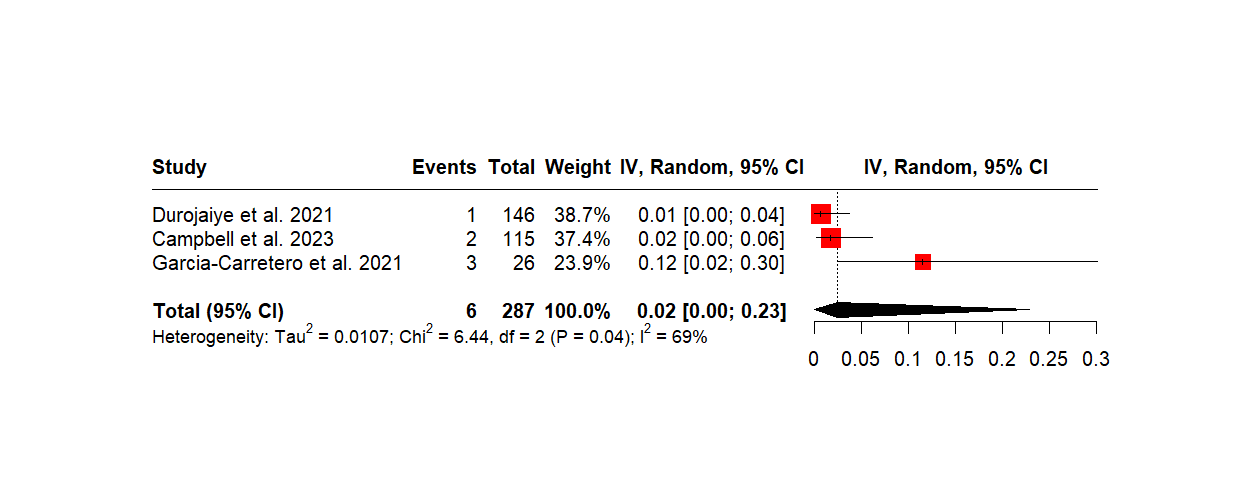


**Supplementary Figure 15:** Forest plot showing the incidence of valvular regurgitation in patients treated with OPAT.

**
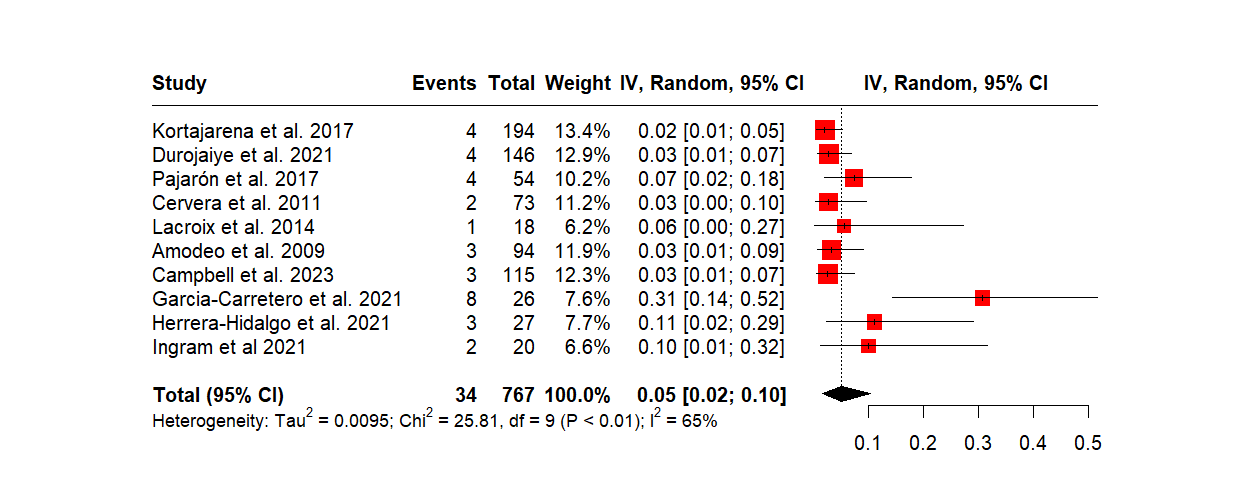
**

**Supplementary Figure 16**: Forest plot showing the incidence of heart failure in patients treated with OPAT.


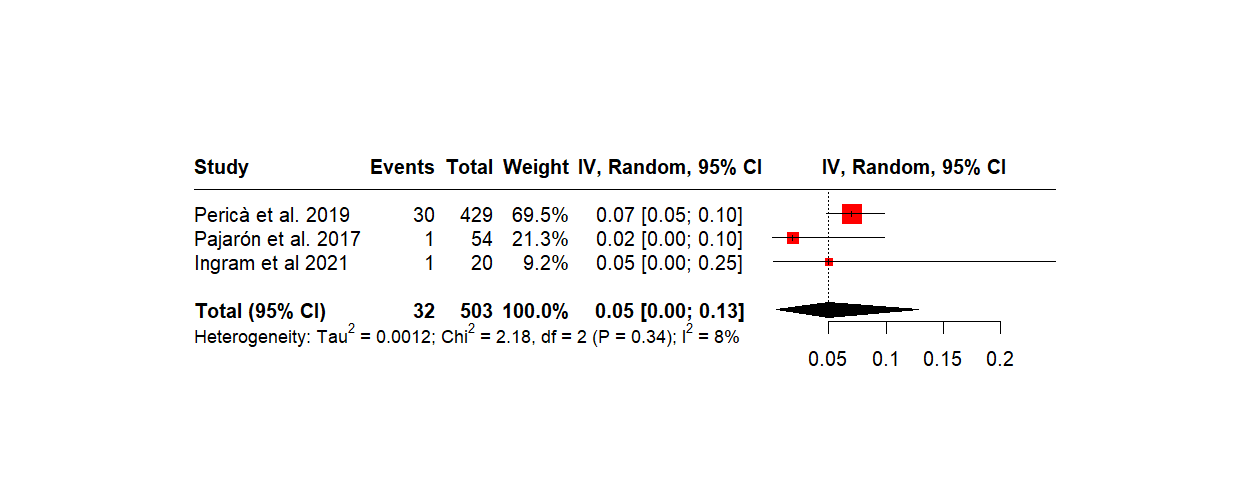


**Supplementary Figure 17:** Forest plot showing the incidence of arrhythmia in patients treated with OPAT.


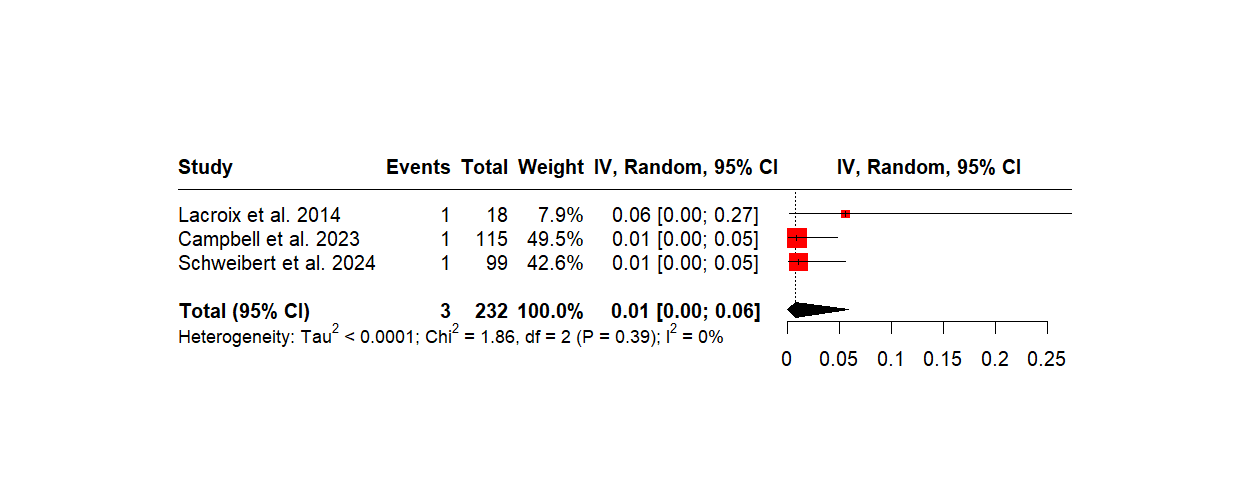


**Supplementary Figure 18**: Forest plot showing the incidence of stroke in patients treated with OPAT.


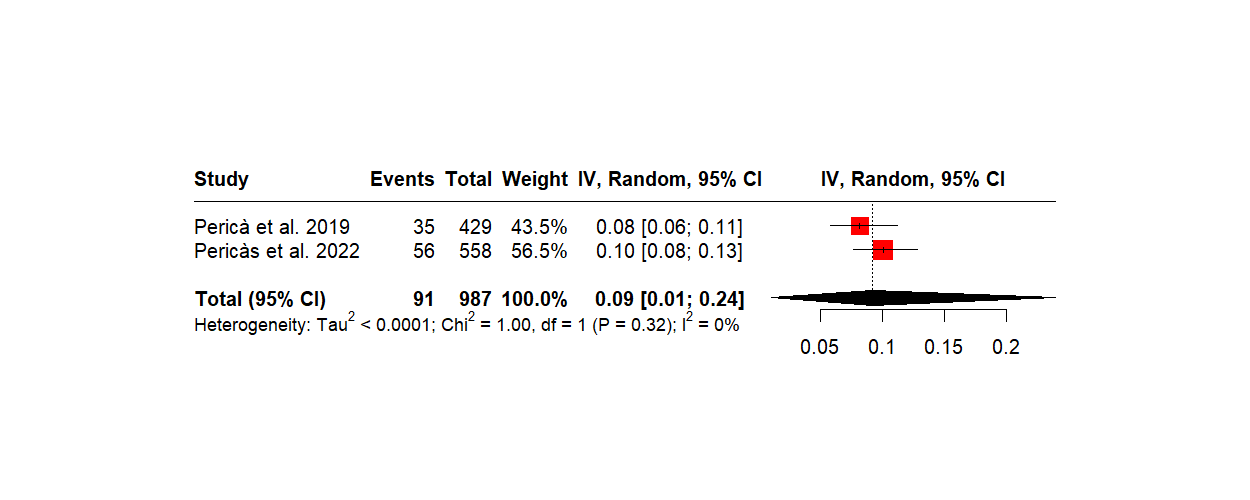


**Supplementary Figure 19:** Forest plot showing the incidence of persistent bacteremia in patients treated with OPAT.


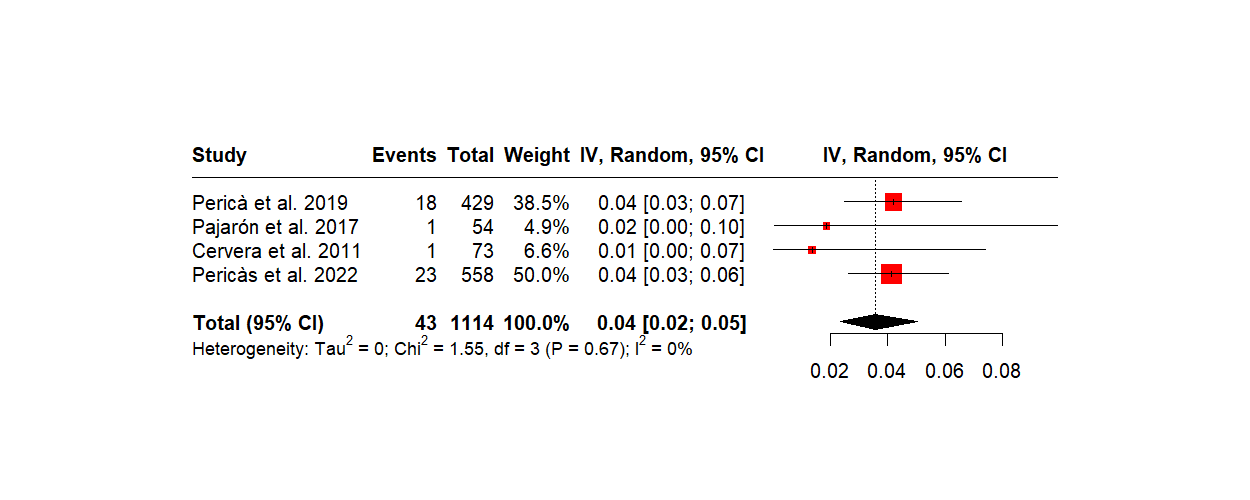


**Supplementary Figure 20:** Forest plot showing the incidence of sepsis in patients treated with OPAT.


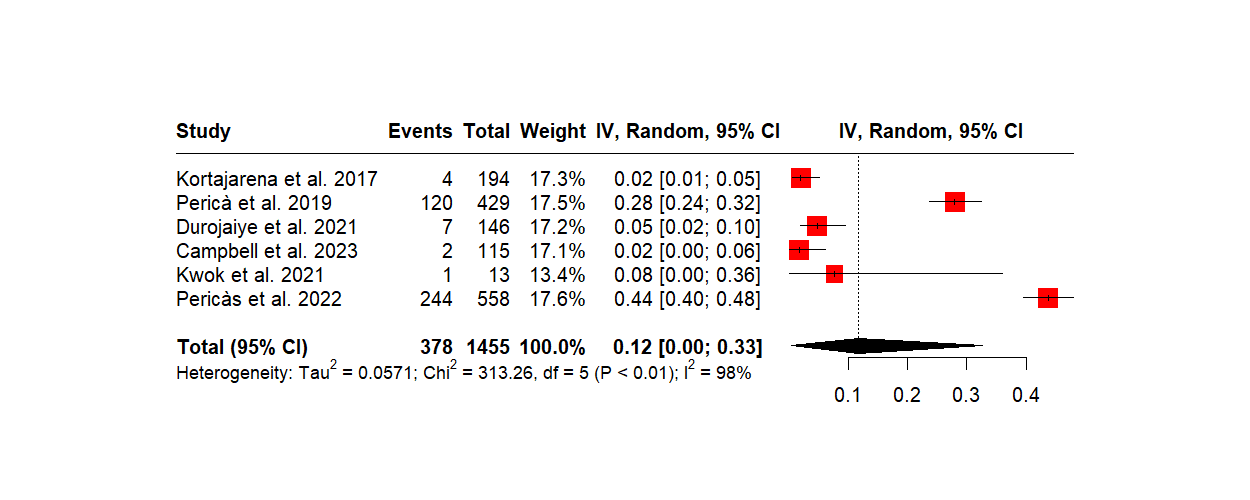


**Supplementary Figure 21**: Forest plot showing the incidence of embolic complications in patients treated with OPAT.


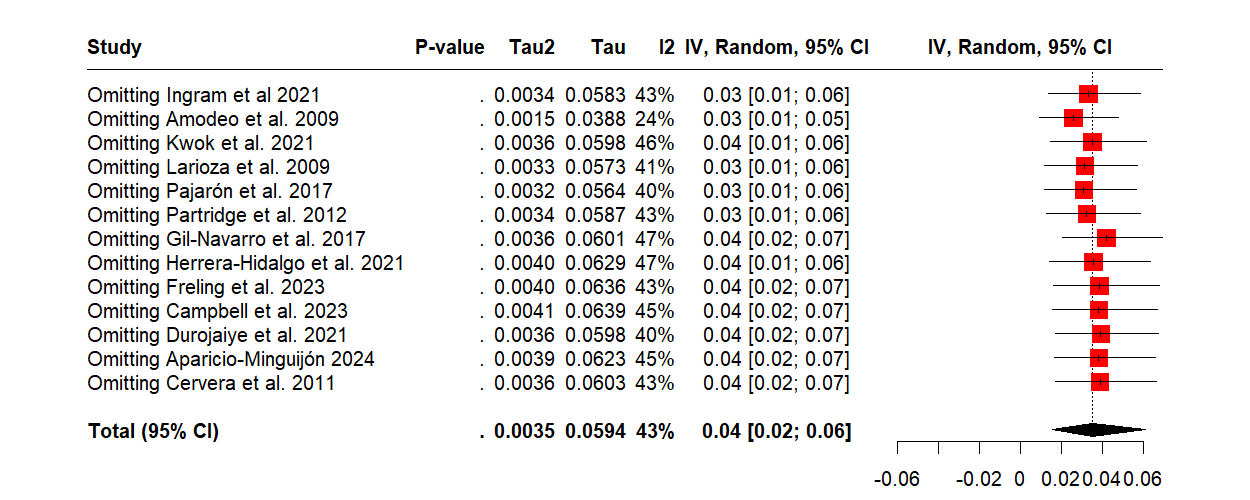


**Supplementary Figure 22**. Sensitivity analysis for drug allergy or complication.


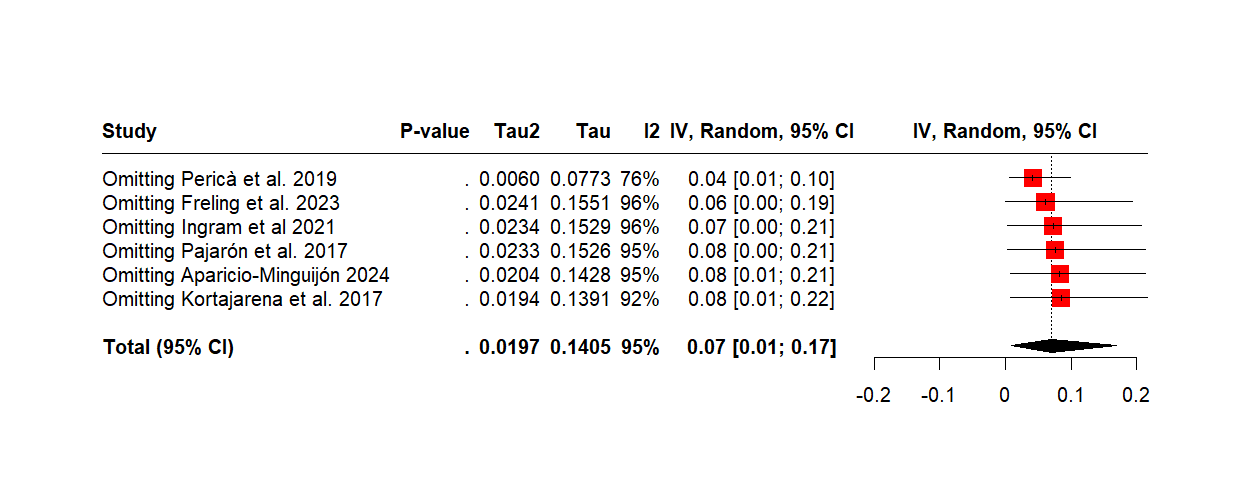


**Supplementary Figure 23**. Sensitivity analysis for acute kidney injury or failure.


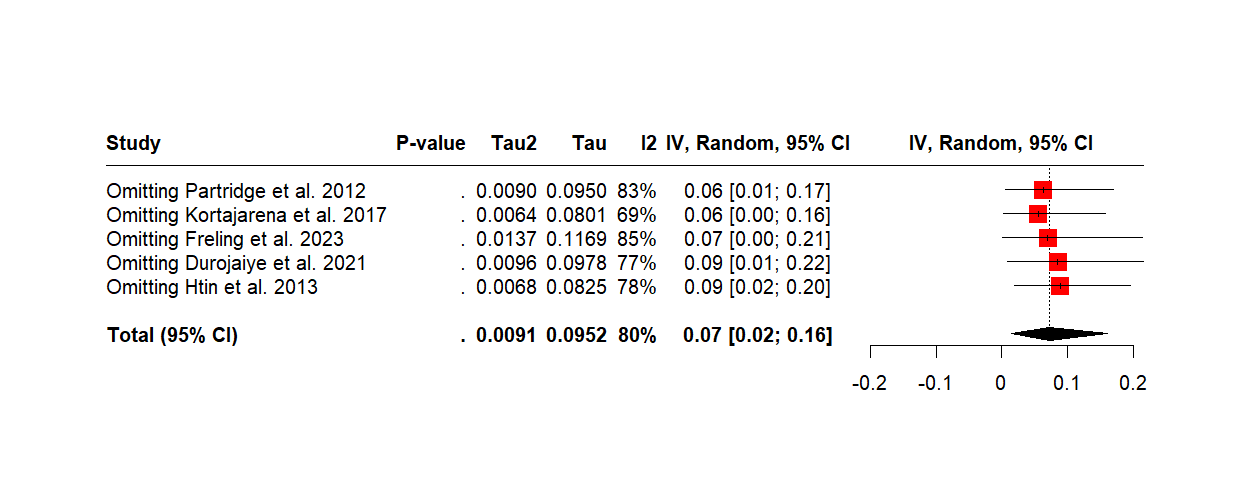


**Supplementary Figure 24**. Sensitivity analysis for IV-line related adverse events.


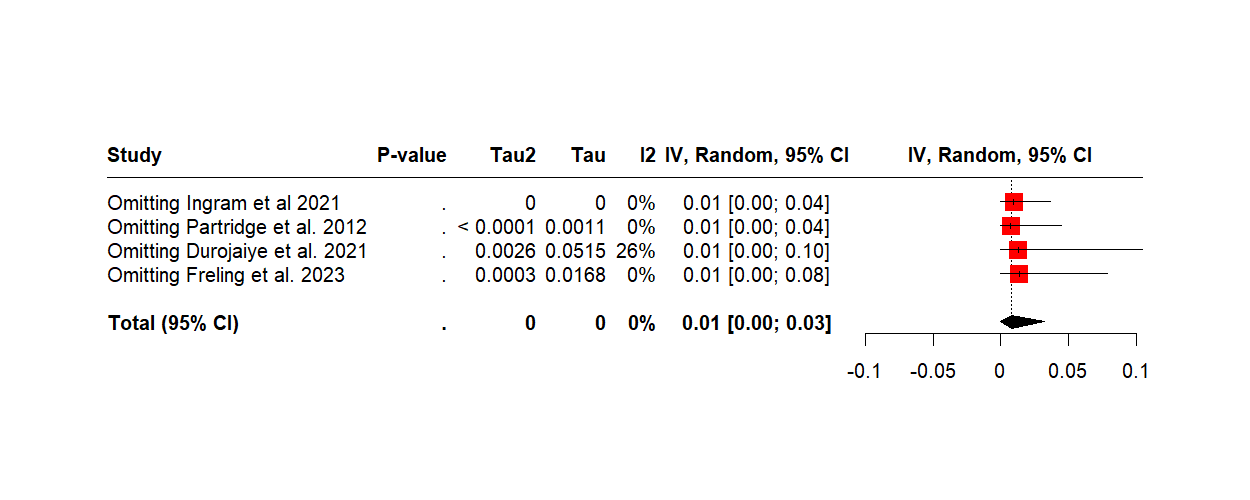


**Supplementary Figure 25**. Sensitivity analysis for *Clostridioides difficile* colitis.


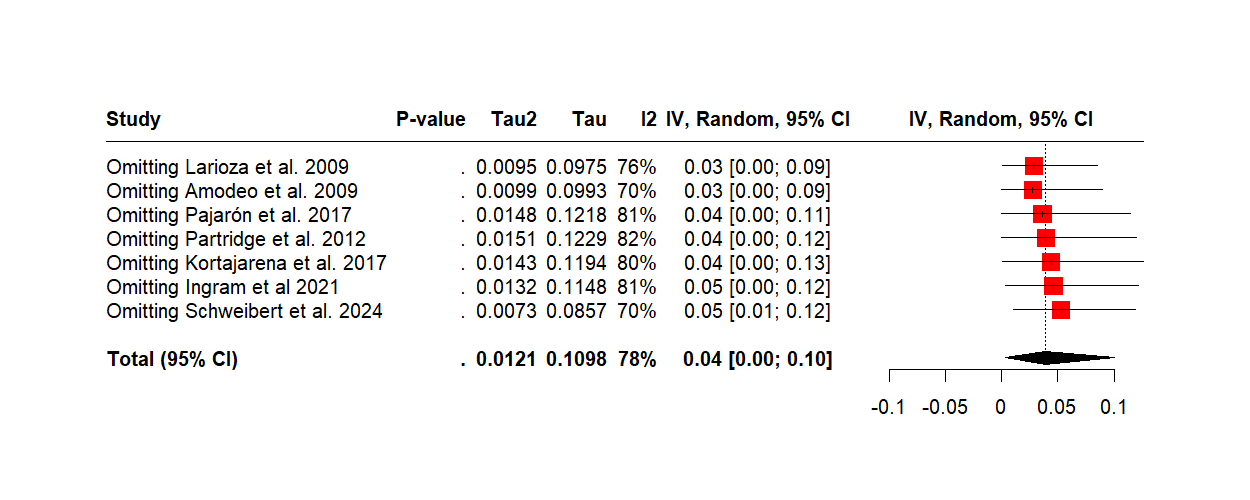


**Supplementary Figure 26**. Sensitivity analysis for PICC line complications.


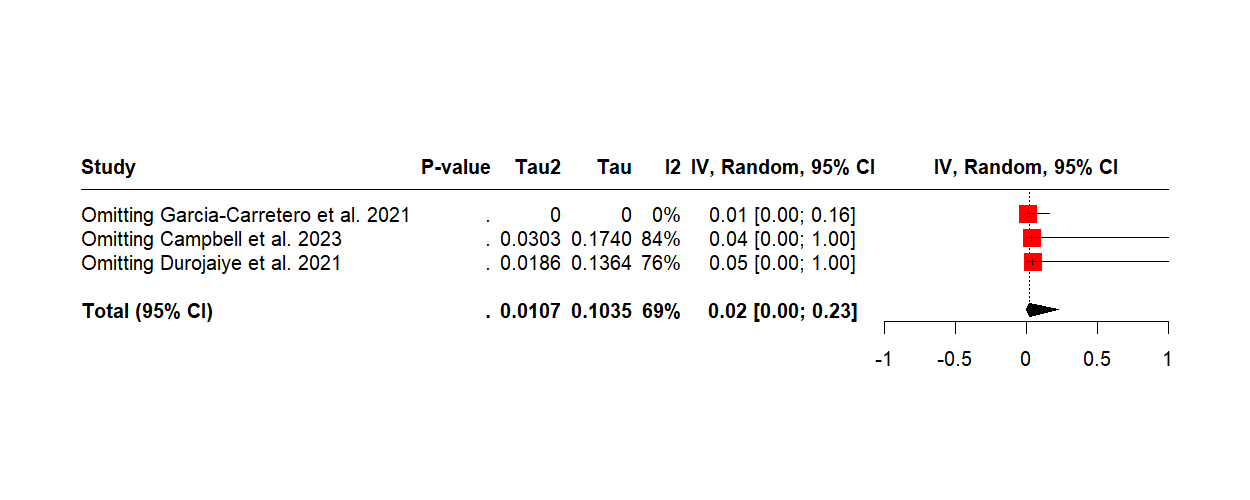


**Supplementary Figure 27**. Sensitivity analysis for valvular regurgitation.


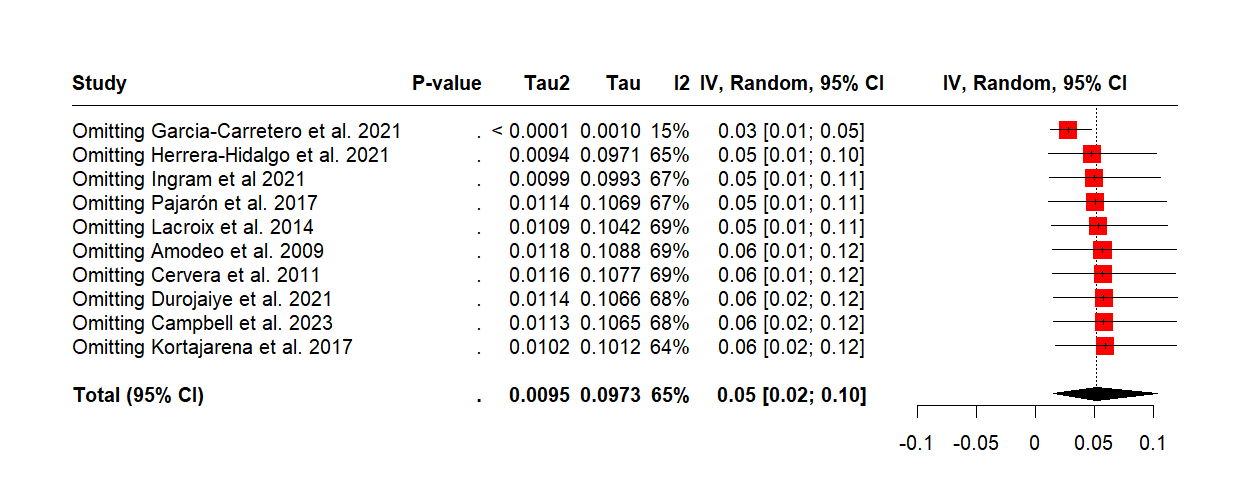


**Supplementary Figure 28**. Sensitivity analysis for heart failure.


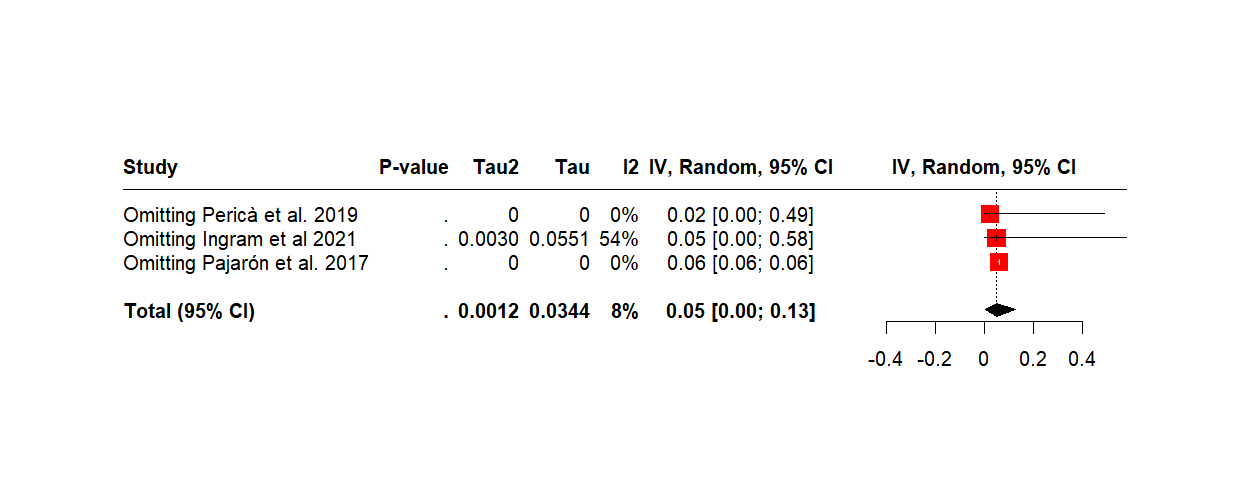


**Supplementary Figure 29**. Sensitivity analysis for arrhythmia.


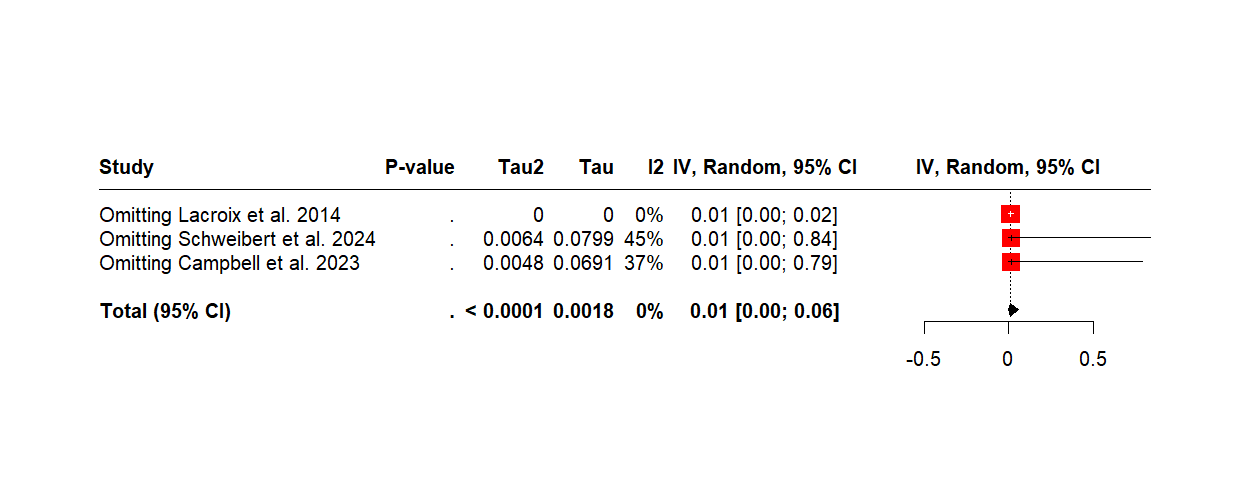


**Supplementary Figure 30.** Sensitivity analysis for stroke.


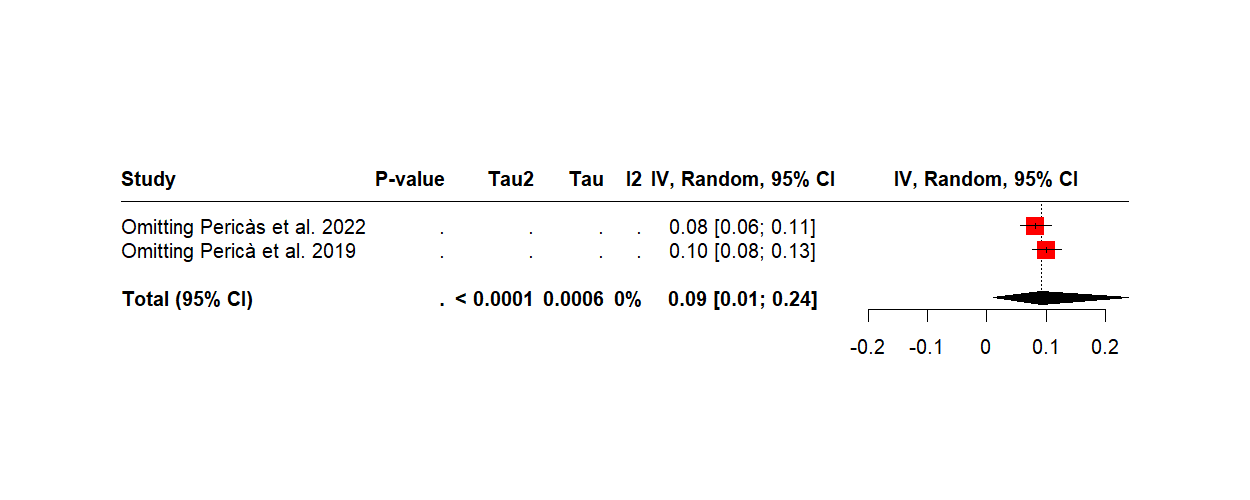


**Supplementary Figure 31**. Sensitivity analysis for persistent bacteremia.


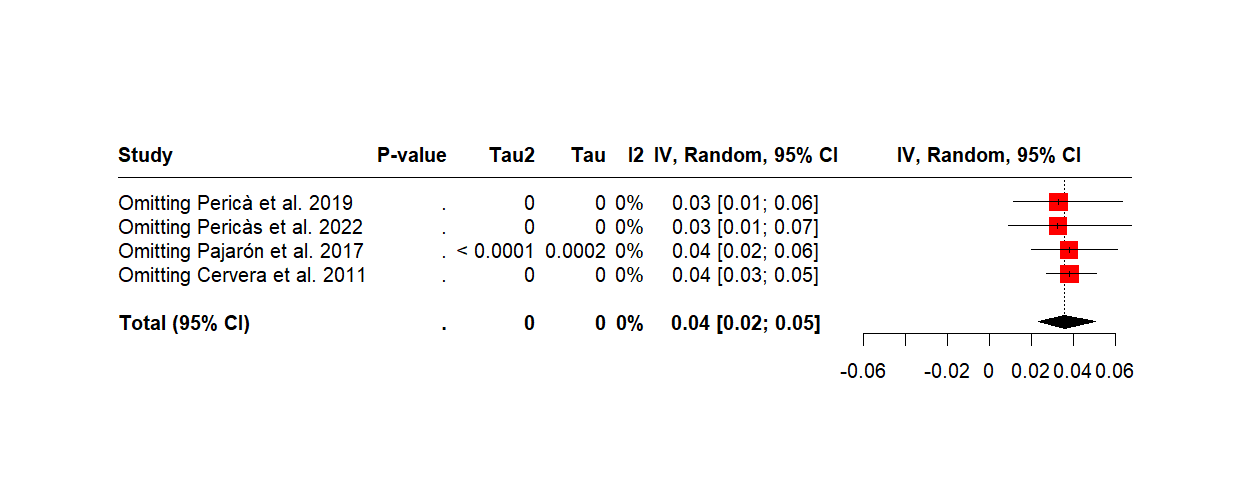


**Supplementary Figure 32**. Sensitivity analysis for sepsis.


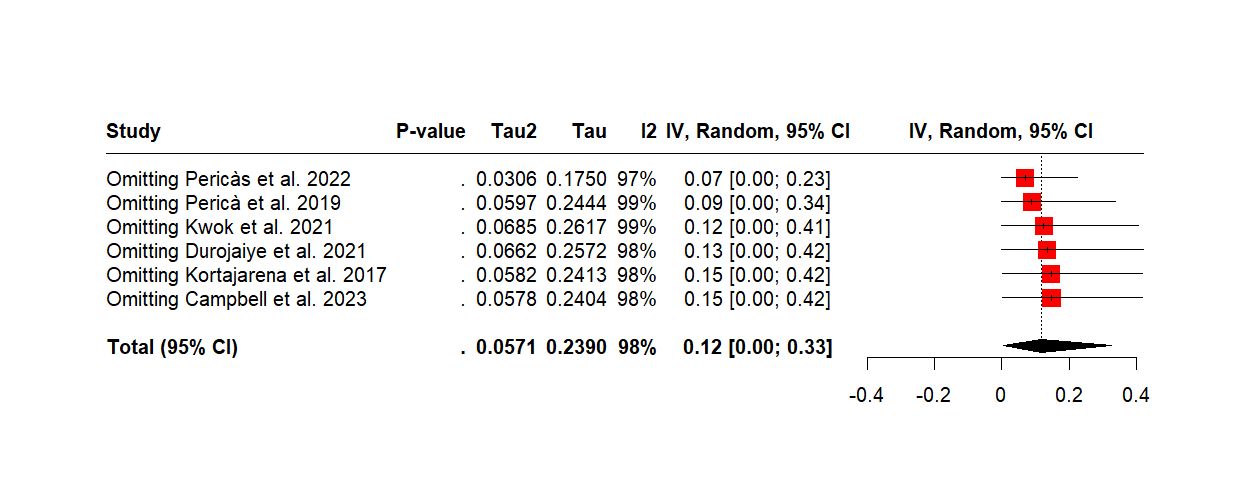


**Supplementary Figure 33**. Sensitivity analysis for embolic complications.


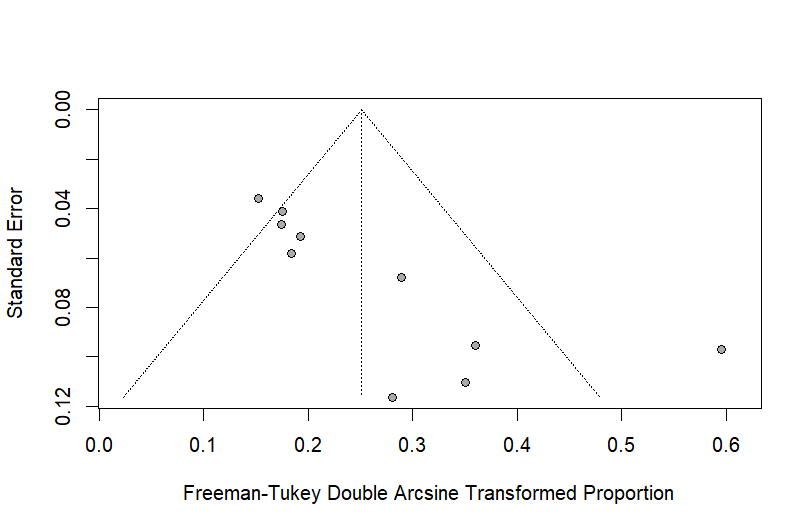


**Supplementary Figure 34**. Funnel plot for publication bias in patients with heart failure.


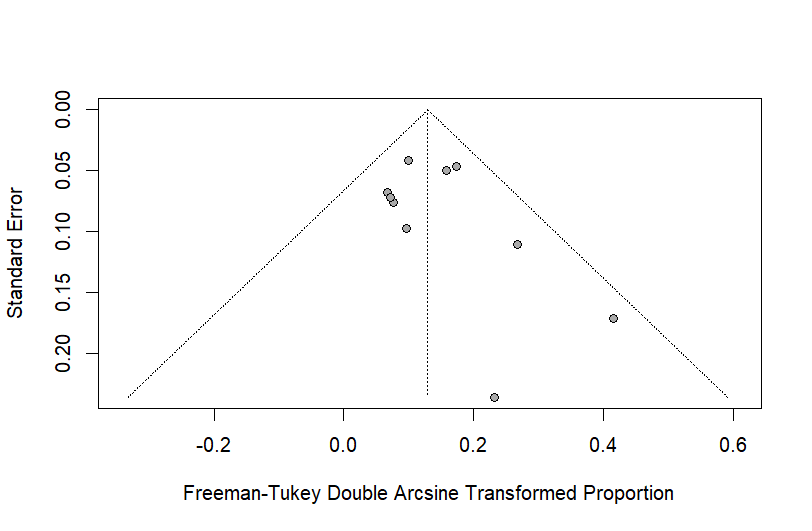


**Supplementary Figure 35**. Funnel plot for publication bias in mortality rate during the treatment period.


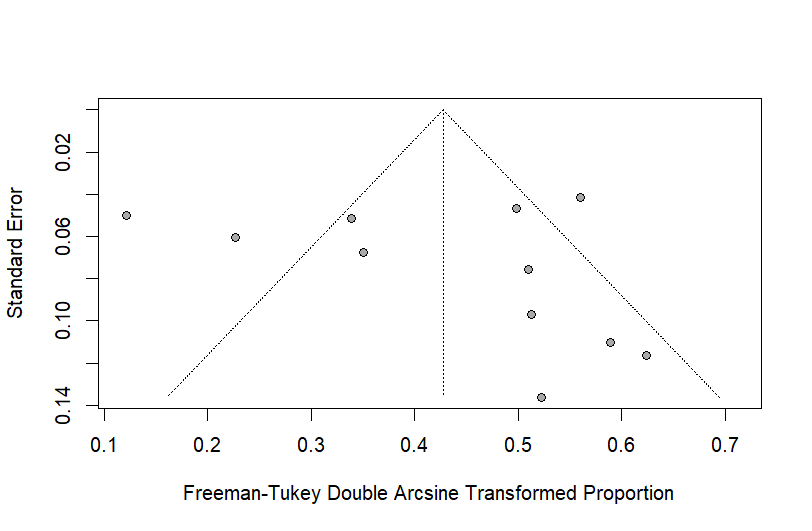


**Supplementary Figure 36**. Funnel plot for publication bias in readmission rate during the treatment period.


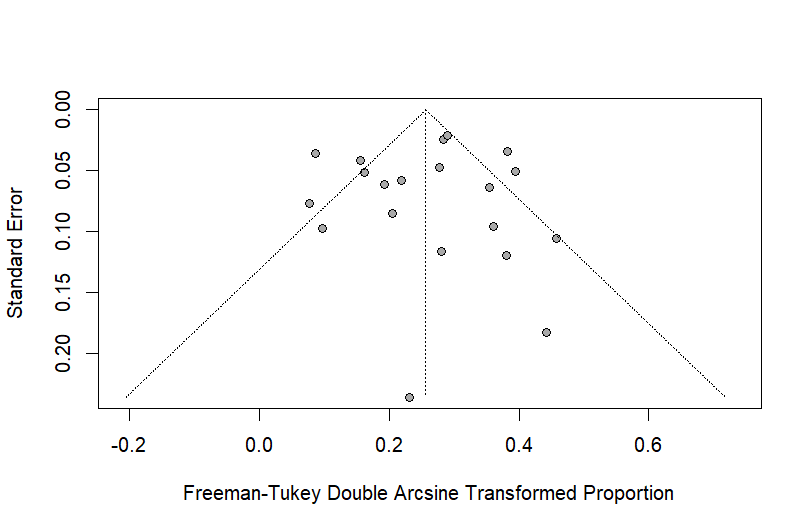


**Supplementary Figure 37**. Funnel plot for publication bias in mortality rate during the follow-up period.


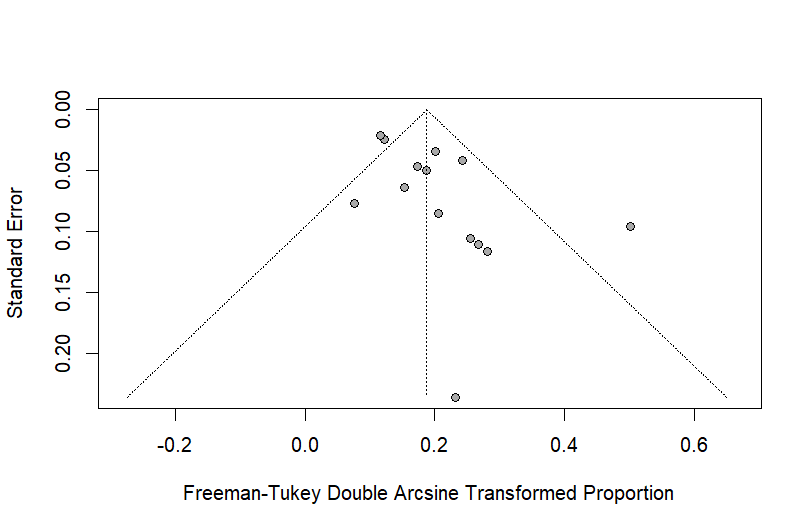


**Supplementary Figure 38**. Funnel plot for publication bias in relapse rate during the follow-up period.


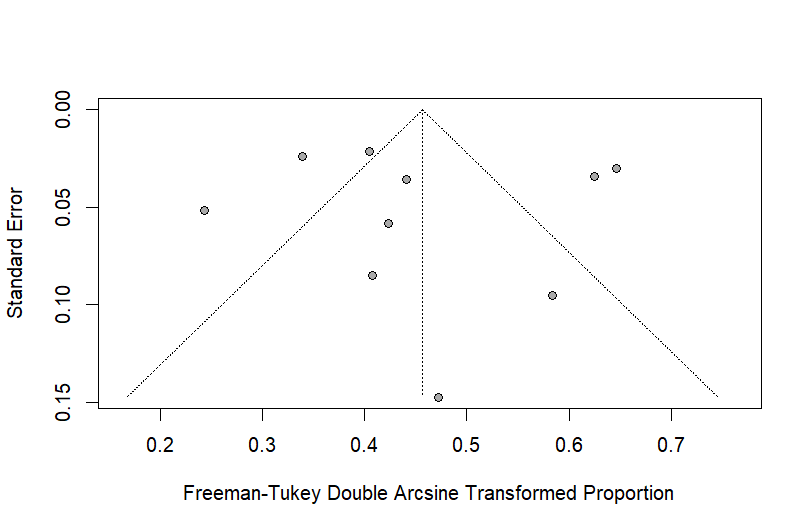


**Supplementary Figure 39**. Funnel plot for publication bias in readmission rate during the follow-up period.


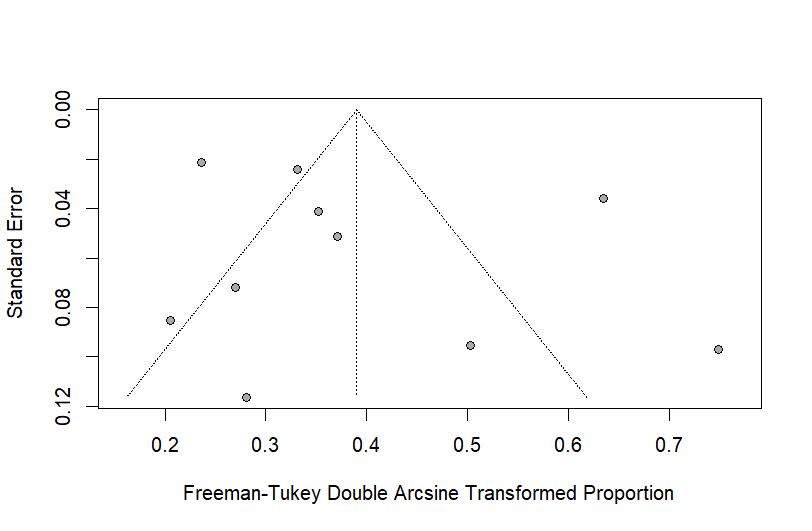


**Supplementary Figure 40**. Funnel plot for publication bias in patients requiring valve replacement or cardiac surgery during the follow-up period.


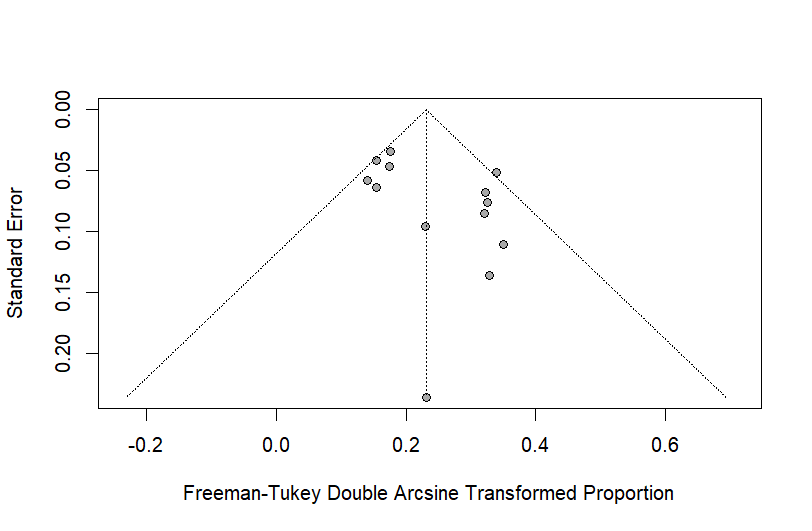


**Supplementary Figure 41**. Funnel plot for publication bias in patients with a drug allergy or complication.
